# Metastatic Transition of Pancreatic Ductal Cell Adenocarcinoma Is Accompanied by the Emergence of Pro-Invasive Cancer-Associated Fibroblasts

**DOI:** 10.3390/cancers14092197

**Published:** 2022-04-28

**Authors:** Shaofei Liu, Yasir Suhail, Ashkan Novin, Lorrie Perpetua, Kshitiz

**Affiliations:** 1 Department of Biomedical Engineering, University of Connecticut Health, Farmington, CT 06030, USA; shaofei.liu_sr@uconn.edu (S.L.); yasir.suhail@uconn.edu (Y.S.); novin@uchc.edu (A.N.); 2 Center for Cell Analysis and Modeling, University of Connecticut Health, Farmington, CT 06030, USA; 3 Research Tissue Repository, University of Connecticut Health, Farmington, CT 06030, USA; lperpetua@uchc.edu

**Keywords:** cancer-associated fibroblasts, pancreatic ductal cell carcinoma, ELI, PDAC, fibroblast, stromal invasibility, cancer stroma, cancer dissemination, initiation of metastasis, cancer stroma interaction

## Abstract

**Simple Summary:**

How stromal cells control tumor progression is an active area of investigation. Cancer associated fibroblasts (CAFs) have been suggested to both limit, or promote cancer dissemination in various contexts, but a systemic understanding of stromal influence in determining cancer metastasis is lacking. This study takes a closer look at the diversity of fibroblasts that emerge in different metastatic stages of PDAC (Pancreatic Ductal Cell Adenocarcinoma), a cancer type with high stromal infiltration. Specifically, we found that the CAFs respond to the growing (but yet not metastasized) tumor by transition to a myofibroblast like stage, potentially resisting cancer invasion. As the cancer disseminates, these fibroblasts are nearly entirely replaced by highly diverse set of subpopulations with distinct functions. We have previously advanced a framework, termed Evolved Levels of Invasability (ELI), explaining the vast differences in cancer metastasis and placental invasion among mammals. We found that PDAC’s metastatic transition and stromal trespass is accompanied by the downregulation of pro-resistive and the upregulation of pro-invasability ELI signatures in CAF subpopulations.

**Abstract:**

Cancer-associated fibroblasts (CAFs) are now appreciated as key regulators of cancer metastasis, particularly in cancers with high stromal content, e.g., pancreatic ductal cell carcinoma (PDAC). However, it is not yet well understood if fibroblasts are always primed to be cooperative in PDAC transition to metastasis, if they undergo transformation which ensures their cooperativity, and if such transformations are cancer-driven or intrinsic to fibroblasts. We performed a fibroblast-centric analysis of PDAC cancer, as it transitioned from the primary site to trespass stromal compartment reaching the lymph node using published single-cell RNA sequencing data by Peng et al. We have characterized the change in fibroblast response to cancer from a normal wound healing response in the initial stages to the emergence of subclasses with myofibroblast and inflammatory fibroblasts such as signatures. We have previously posited “Evolved Levels of Invasibility (ELI)”, a framework describing the evolution of stromal invasability as a selected phenotype, which explains the large and correlated reduction in stromal invasion by placental trophoblasts and cancer cells in certain mammals. Within PDAC samples, we found large changes in fibroblast subclasses at succeeding stages of PDAC progression, with the emergence of specific subclasses when cancer trespasses stroma to metastasize to proximal lymph nodes (stage IIA to IIB). Surprisingly, we found that the initial metastatic transition is accompanied by downregulation of ELI-predicted pro-resistive genes, and the emergence of a subclass of fibroblasts with ELI-predicted increased invasibility. Interestingly, this trend was also observed in stellate cells. Using a larger cohort of bulk RNAseq data from The Cancer Genome Atlas for PDAC cancers, we confirmed that genes describing this emergent fibroblast subclass are also correlated with lymph node metastasis of cancer cells. Experimental testing of selected genes characterizing pro-resistive and pro-invasive fibroblast clusters confirmed their contribution in regulating stromal invasability as a phenotype. Our data confirm that the complexity of stromal response to cancer is really a function of stage-wise emergence of distinct fibroblast clusters, characterized by distinct gene sets which confer initially a predominantly pro-resistive and then a pro-invasive property to the stroma. Stromal response therefore transitions from being tumor-limiting to a pro-metastatic state, facilitating stromal trespass and the onset of metastasis.

## 1. Introduction

The understanding of cancer metastasis as a cell autonomous disease of tumor cells has changed in the last decade to being a collective outcome of cancer cells and the acellular and cellular constituents of the tumor microenvironment [1]. Although the genetic and the developmental trajectories of tumor cells play primary roles in determining the progress of cancer through the metastatic cascade, it is now well appreciated that other cell types, particularly fibroblasts and macrophages, cooperate with cancer cells through the various steps involved in metastasis. The first step in cancer metastasis is stromal dissemination, wherein either mesenchymal or clusters of epithelial-like cancer cells break through the basal lamina into the stromal tissue [2]. The first line of non-immunogenic defense against cancer dissemination is therefore the stromal fibroblasts surrounding the primary lesion. It is however not properly understood whether tissue resident fibroblasts generally resist cancer invasion and if the activation of these fibroblasts to physiologically respond to the primary lesion always acts in favor of cancer dissemination [3,4,5,6]. Are there consistent paradigms in the role of tissue-resident fibroblasts and cancer-associated fibroblasts (CAFs) in regulating cancer dissemination which could potentially be harnessed to limit early cancer dissemination? 

We have previously advanced an evolutionary framework, termed the Evolved Levels of Invasibility (ELI), explaining the large differences in the rate of cancer metastasis across mammals, which also correlates with the degree of their placental invasion into the endometrial stroma during pregnancy [7]. ELI posits that mammals with non-invasive placentation have evolved resistive stromal mechanisms to limit trophoblast invasion from the placenta [8]. Secondary manifestation of this phenomenon results in much more resistive stroma, resulting in the containment of cancer within its primary site preventing dissemination and metastasis [7,9]. We have shown that for various cancers, in particular melanoma, these ELI signatures are highly enriched in human cancers [10], indicating that knowledge gained from comparative fibroblast biology across species can provide useful insights into the mechanisms driving fibroblastic response to human cancers. ELI posits that stromal invasability can be considered as a regulated phenotype, which has resulted in dramatically different levels of stromal resistance to epithelial invasion across eutherian species [7]. 

Cell-type-specific data within cancers are now beginning to be collected, both at the transcriptomic level by single-cell RNA sequencing (scRNAseq) and single-cell proteomics [11,12,13]. However, the current state of clinical data is still limited at the single-cell level, requiring sophisticated deconvolution approaches to delineate the stromal compartment in bulk RNAseq data, in order to investigate the contribution of stromal cells in cancer progression from large amounts of vertical patient data [14,15]. We therefore chose to explore the role of fibroblasts in regulating early cancer dissemination in recently published scRNAseq datasets and specifically test the presence of ELI signature in fibroblasts in the early stages of cancer metastasis. Because pancreatic cancers are associated with a relatively large stromal component in its cellular composition [6,16], we explored the transcriptomic state of fibroblasts in a moderately large study of pancreatic ductal adenocarcinoma (PDAC) patients at different stages of cancer metastasis [17]. PDAC is marked by high intra-tumoral heterogeneity, even within stromal cells, and has very poor prognosis [16,18,19]. 

We identified remarkable changes in the fibroblast signaling state during early cancer dissemination, marked by an initial fibroblastic activation responding to the growing primary lesion, followed by a sudden increase in fibroblast heterogeneity as cancer disseminates to neighboring lymph nodes. Recently, using transcriptomic data from endometrial fibroblasts of many mammalian species, we and our collaborators have identified genes and transcription factors that have correlatively changed according to their levels of invasibility [20]. Crucially, we also found that this early dissemination is marked by a large and significant downregulation of pro-resistive ELI^dn^ genes, which are highly expressed in epitheliochorial species (e.g., cows), similar to our previous findings from bulk data in melanoma [10]. Furthermore, we also found that the transition to metastasis is accompanied by the emergence of a fibroblast subclass with enrichment of pro-invasable ELI^up^ genes, expressed in hemochorial species (e.g., humans and rodents) [20]. We confirmed these trends in a larger cohort of PDAC patient data from The Cancer Genome Atlas (TCGA) in early metastatic stages. Overall, our study presents a detailed fibroblast-centered understanding of pancreatic cancer’s progress in the metastatic cascade, providing useful insights in the emergence of pro-invasable fibroblast subpopulations, identifying potential opportunities to target the stromal microenvironment to limit the early spreading of cancer.

## 2. Materials and Methods

### 2.1. Single-Cell Data

The multi-tumor single-cell RNAseq data in pancreatic adenocarcinoma were obtained from Peng et al. [17]. 

### 2.2. Single-Cell RNA Sequencing Processing

We used Seurat (v3.1.5, Rahul Satija Lab, New York University, New York, NY, USA) for all cell clustering and differential expression analysis between cell clusters. Tumor and normal samples were co-clustered together, using the method of Stuart et al. [21]. For clustering all the cells, the top 10 Principal Component Analysis (PCA) dimensions were used to assign k-nearest neighbors and for downstream t-SNE analysis. Cell clusters were identified using t-SNE in Seurat using resolution = 1. Cell clusters obtained above were assigned biological identities following the markers from Peng et al. (e.g., LUM, DCN, and COL1A1 expression were used to identify fibroblasts). In total, 12 cell types were identified, Endothelial cell, Ductal cell 1, Stellate cell, Fibroblast, Ductal cell 2, Acinar, Macrophage, Endocrine cell, B cell, T cell, Ductal cell 1 + Ductal cell 2 (the clusters contain markers of both cell types), and others (no cell makers we are interested). For clustering the cell subtypes within fibroblasts, after PCA, the top 7 principal components were selected using Seurat’s Elbow plot for downstream t-SNE. Cluster-specific genes and differential expression between clusters were performed using Seurat’s FindMarkers function.

### 2.3. Receptor–Ligand Processing

Receptor–ligand partners were obtained from Ramilowski et al. [22]. The strength of a ligand–receptor strength between cell types was computed as the product of the ligand expression, receptor expression, the fraction of receiving cells expressing the receptor gene, and the fraction of secreting cells expressing the ligand gene. In the bubble plots, the interactions were selected by including the top 4 ligand/receptors expressed by the fibroblasts and top 3 partner genes expressed by the other cell type. Circos plots were produced using Krzywinski et al. [23]. 

### 2.4. Pathway Enrichment and Evolved Levels of Invasability

The top genes aligning to endometrial invasability were obtained using the linear regression in Suhail et al. [10,20]. Gene set enrichment analysis was performed using fgsea package in R [24]. Marker genes were selected using a cutoff of *p* value < 0.05, and abs(log2FoldChange) > 0.5 for further processing. Upstream regulators and transcription factors were obtained using IPA using the above thresholds for DE genes. All GO and KEGG pathway enrichments were calculated using ClusterProfiler 4.0 [25]. ELI^dn^ and ELI^up^ subnetworks were computed by including protein interactions from Reactome [26]. Gene–gene correlations were computed as the Pearson correlation of the expression of a pair of genes across a cell cluster population. The overall strength of the gene correlations in the ELI^up^ or ELI^dn^ gene set was compared with all the gene pairs for all genes outside the ELI^up/dn^ set using a t test. Short Time-series Expression Mining (STEM analysis) was used to find statistically significant patterns of gene expression changes across stages [27]. 

### 2.5. Human Tissue Sourcing and Immunostaining

Optimal cutting temperature (OCT) compound-embedded human pancreatic ductal adenocarcinoma samples were obtained from UConn Health Pathology Biorepository. For representative PDAC samples at pre-metastatic stage IIA, and at the onset of lymph metastasis characterized by stage IIB, we obtained a T2N0Mn/a sample and a T3N1Mn/a sample, respectively, isolated from patients after Whipple resection. The N0 sample was obtained from a de-identified 59-year-old African American, with a tumor size of 3.2 × 3.0 × 2.8 cm with G1 histological grade. From 9 examined lymph nodes, none were found to be positive, suggesting a non-metastatic stage. N1 samples were obtained from a de-identified 67-year-old Caucasian male. From the 13 nodes examined, 5 were involved with metastatic carcinoma. 

OCT-embedded samples were cryosectioned on glass slides and fixed using 4% paraformaldehyde in PBS solution for 20 min. After washing with PBS twice, samples were permeabilized using 0.2% Triton X-100 for 20 min and washed again with PBS. Thereafter, sections were blocked using blocking buffer (10% goat serum in PBS and 0.1% Triton X-100) for 4 h and incubated with primary antibody in blocking buffer overnight at 4 °C. Unbound primary antibody was washed with PBS 4 times, samples were reblocked using blocking buffer for 1 h, and incubated with Alexa-fluor 488 conjugated secondary antibody in blocking buffer for 1 h. After incubation, samples were washed with PBS 4 times, and were mounted on glass coverslips with antifade solution and imaged. 

Imaging was performed on Zeiss Observer Z1 microscope with Colibri.2 LED-based fluorescence and Apotome.2 sectional illumination using a 20× objective. 

Primary antibodies used were: anti-phospho-β-catenin (Ser675) D2F1 (Cell Signaling, Danvers, MA, USA, 4176T) and anti-HIF-1a (Proteintech, Rosemont, IL, USA, 20960). 

### 2.6. Accelerated Nanopatterned Stromal Invasion Assay

ANSIA platform was fabricated using methods previously described [20]. BJ and A375 cells were used to measure stromal invasability in response to stromal genetic perturbation. BJ cells were subjected to CRISPR/Cas9 mediated gene silencing using gRNA from Integrated DNA Technologies (IDT) using CRISPRMAX reagent and used 2 days after transfection for experimentation. Zeiss Observer Z1 was used to perform epifluorescence and phase-contrast microscopy with tri-gas incubators and multi-location scanning stage with 130 × 100 steps, and Definite Focus v2. 10x/0.8 WD-0.55 EC Plan-Apochromat objective was used for imaging at a time interval of 1 h for the total duration of 24 h. 

### 2.7. Quantification of Invasion Characteristics by Invading Cancer Cells into Stroma

Invading cancer cells were imaged using live epifluorescence microscopy every 1 h for 24 h. Invasion fronts were tracked using Region of Interest (ROI) panel in the Fiji software. Underlying anisotropic nanotextured resulted in highly directional invasive fronts, both accelerating the phenomenon, as well reducing dimensionality of the data. ROIs were determined by manual tracing of boundaries, and the normalized extent of invasion determined by dividing total *δArea* by the length of the initial cancer–stroma interface.

$$\delta Area\left(t\right)=Area\left(t\right)-Area\left(t0\right)$$

$$\langle \delta Area\left(t\right)\rangle =\frac{\delta Area\left(t\right)}{L\left(Interface\right)}$$

ROIs were converted to a one-dimensional profile of the invasive front, which was smoothed by moving the average over 20 pixels. Smoothed profile was used to calculate the mean signal to either side (40 pixel each) of every point, and a peak was determined when the smoothed profile was larger than either side averages. Consecutive peaks emerged and were determined at the midpoint of the interval. 

## 3. Results

**Figure 1:**
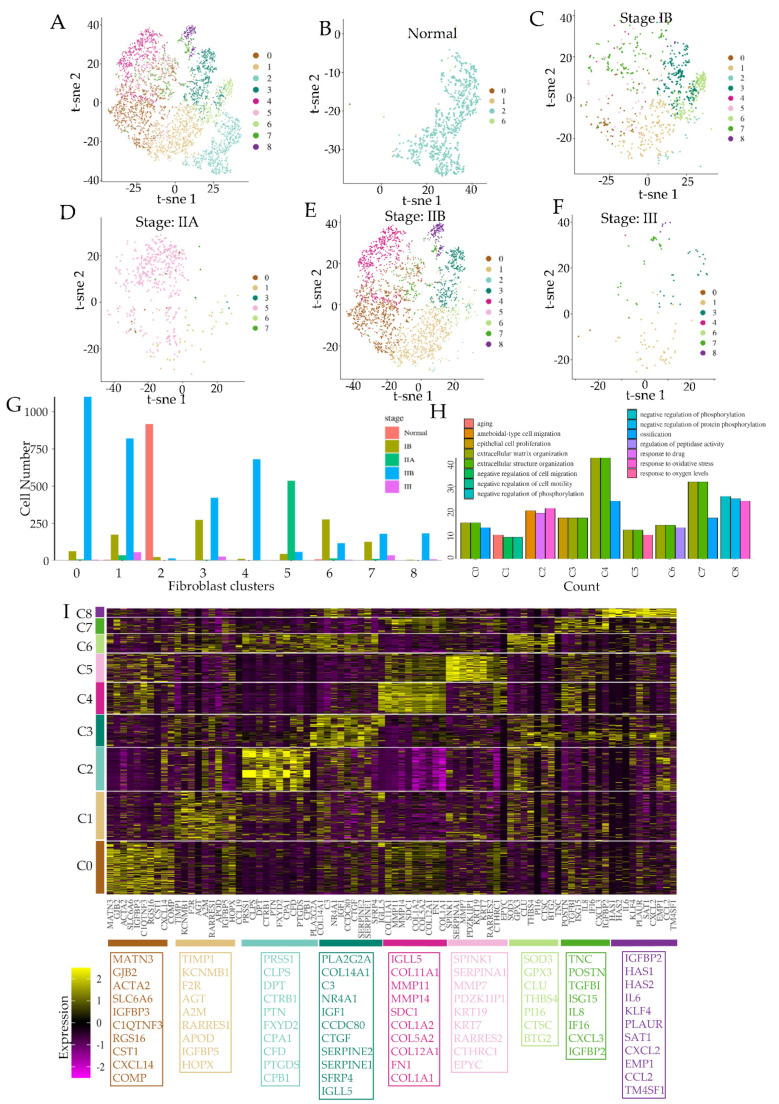
Metastatic progression of pancreatic ductal cell carcinoma (PDAC) is associated with dynamic emergence of fibroblast subclasses with distinct transcriptional profiles. (**A**–**F**) Fibroblasts exhibit dramatic changes in transcriptomic diversity across progressive stages of PDAC cancer. (**A**) t-sne representation of fibroblasts from all pooled samples of PDAC cancers as well as normal pancreas [17] clustered to highlight their transcriptomic differences; t-sne representation of fibroblasts clustered for all pooled samples, and shown for (**B**) normal, (**C**) stage IB (T1N0M0) with tumor size < 2 cm, (**D**) IIA (T2 N0M0) with tumor size < 4 cm, (**E**) IIB (T1/2N1M0) with lymph metastasis < 3 nodes, and (**F**) III (T1/2/3N1/2/M1) with distal metastasis; Quantification of the proportion of fibroblast clusters at different stages of PDAC in (**G**). (**H**) Gene ontology activation scores in different fibroblast clusters; each term is obtained by differential gene expression analysis (log2fc ≥ 0.5, *p*-value ≤ 0.05). (**I**) Heatmap showing top 10 genes as markers for each fibroblast cluster, compared to all fibroblasts; colors to the left and the bottom correspond to the fibroblast clusters identified in t-sne representation in A.

To specifically explore the state of fibroblasts in normal and cancerous PDAC tissues, we used previously published scRNAseq data obtained from 35 patients (24 primary PDAC tumors, 11 control pancreas), identified with the stages of the cancer progression [17]. We identified fibroblasts (see Methods) and explored their diversity in all samples, as well as in samples categorized according to cancer progression stages. The AJCC (American Joint Committee on Cancer) prescribed TNM system describes the metastatic stage of PDAC cancers according to tumor size (T), lymph node spread (*N*), and distant metastasis (M) [28]. t-sne analysis for all pooled samples showed a remarkable diversity in fibroblasts, clustered in eight different groups according to their global transcriptomic profiles (Figure 1A). However, when we observed the fibroblasts assigned to their clusters in the pooled data at different stages in cancer, we found remarkable changes in transcriptomic diversity. While fibroblasts from the normal pancreas (*n* = 11 patients) were homogenous and largely constituting a single cluster (cluster 2) (Figure 1B), non-metastatic growth of tumor in stage IB (T2N0M0) resulted in the emergence of new fibroblast-specific clusters (Figure 1C). As the tumor size grew in stage IIA (T3N0M0) without lymph node metastasis, the diversity in fibroblasts decreased to predominantly a single cluster (cluster 5) (Figure 1D). Remarkably, fibroblasts assumed a much more diverse transcriptomic state in stage IIA (T1-3N1M0), which is characterized by the spread of cancer to lymph nodes (Figure 1E). Transition from stage IIA to IIB constitutes the spread of cancer from its initial confines to trespass the stromal compartment reaching the lymph nodes, and a window into this transition can allow us to understand the initial fibroblast response to cancer in its early dissemination stage. This transition was accompanied by a change in the fibroblast population from being very homogeneous to the emergence of many diverse fibroblast subclasses (cluster 0,1,3,4,7,8) (Figure 1D,E). The data for stage III (T1-3N2M0) were sparse, but the overall diversity was retained (Figure 1F). An initial stage-wise analysis of fibroblasts therefore showed dynamic changes in the emergence and reduction of various fibroblast subclasses along the early metastatic cascade (Figure 1G). 

### 3.1. Pancreatic Cancer Transition to Metastasis Is Characterized by the Emergence of Distinct Subclasses of Fibroblasts with Different Phenotypic Roles Supporting Stromal Trespass 

Because fibroblast subclasses in pancreatic ductal cell adenocarcinoma changed dramatically with the emergence, and then the transition of cancer towards metastasis, we explored the gene expression data further within these clusters. Gene ontology (GO) analysis showed that clusters present in pre-metastatic stages had a higher presence of genes belonging to extracellular matrix (ECM) generation, cell–matrix interaction, as well as hypoxia (Figure 1H). Clusters abundant in fibroblasts post-metastasis showed the expression of genes belonging to gene sets associated with secretory pathways, putatively associated with inflammatory signaling and complement activation (Figure 1H and Figure S1). Gene clustering revealed key genes which were enriched in each of the clusters. Cluster 2 for which normal fibroblasts were homogenous for (Figure 1B), were characterized by genes indicating supporting stromal role of pancreatic fibroblasts, particularly encoding many serine peptidases. These include mostly matrix related or other proteases dermatopontin (DPT), colipase (CLPS), trypsin-1 (PRSS1), chymotrypsinogen B1 (CTRB1), and carboxypeptidases A1 (CPA1), B1 (CPB1), and complement factor D (CFD), which is also a member of the chymotrypsin family of serine proteases, as well as other protein coding genes including heparin binding growth factor-like pleiotrophin (PTN). In contrast, cluster 4 was associated with increased expression of genes encoding matrix remodeling constituents, including several matrix metalloproteinases (MMPs) and extracellular matrix components including collagens and fibronectin. Cluster 5, which was what fibroblasts in the pre-dissemination stage IIA were homogenous for, was characterized by inhibitors of protease and peptidase activity, including SPINK1 which encodes a pancreatic secretory trypsin inhibitor, SERPINEA1 encoding alpha-1 antitrypsin, as well as keratins encoding KRT7, and 19. Interestingly, cluster 5 was also enriched for RARRES2 encoding chemerin, an adipokine which could mediate anti-proliferative retinoic acid signaling, as well as MMP7 and collagen triple helix containing protein 1 (CTHRC1) associated with fibrotic processes. Entering into early metastasis in stage IIB is characterized by emergence of cluster 0, 1, 3, and 4. Cluster 0 marked fibroblasts encoding matricellular proteins such as matrillin (MATN3) and cartilage oligomeric matrix protein (COMP), as well as smooth muscle actin 2 (ACTA2) in addition to channel proteins include gap junction beta 2 (GJB2), and SLC6A6, encoding a sodium- and chloride-dependent transporter. Cluster 1 had enriched expression of genes associated with vascular control, including angiotensinogen (AGT), receptor for thrombin (F2R), metallopeptidase inhibitor 1 (TIMP1), and apolipoprotein D precursor (APOD). Cluster 3, also present in fibroblasts at the metastatic stage, was marked by several secreted signaling molecules, including connective tissue growth factor (CTGF), Wnt inhibitor secreted frizzled-related protein 4 (SFRP4), complement factor C3, as well as insulin growth factor (IGF1) and inhibitors of matrix metalloproteinases, SERPINE1/2. Cluster 6 was largely characterized by genes related to reactive oxygen species antioxidant response, including an extracellular superoxide dismutase (SOD3), glutathione peroxidase (GPX3). Cluster 7 showed increased expression of genes encoding tenascin (TNC), periostin (POSTN), transforming growth factor induced (TGFI), as well as secreted factors including interleukin 8 (IL8), CXCL3, and IGFBP2. These genes indicated an advanced desmoplastic response, present in matured fibrosis which are characterized by the overactivation of the TGFβ pathway and secretion of inflammatory cytokines [29]. 

**Figure 2:**
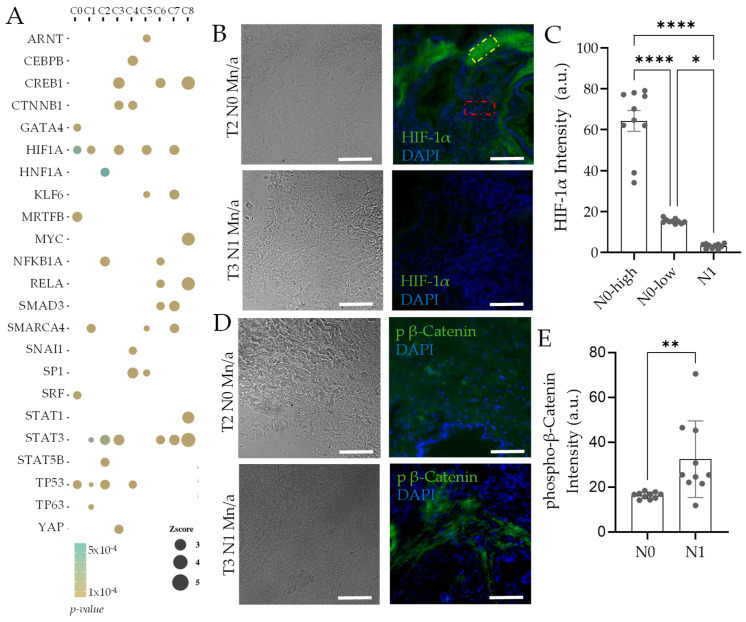
Predicted stage-wise activation of transcription factors in fibroblasts is indicated in patient samples of PDAC cancers. (**A**) Top 5 IPA-predicted transcription factors activators in each fibroblast cluster identified in Figure 1 and their z-score in all other clusters. (**B**–**E**) Immunohistochemistry in patient tumor samples before and after lymph node metastasis (termed N0 and N1, respectively, in TNM nomenclature) for predicted TFs exclusive to C5 and C4 clusters present in respective stages. (**B**) Representative phase and immunofluorescence images showing areas with very high (green square) and moderate fluorescence intensity (red square) in areas stained with HIF-1α antibody in the non-metastasized sample (T2N0), while reduced HIF-1α levels in the patient samples with lymph metastasis (T3N1); Quantification of HIF-1α levels in N1 and N0 samples in spatial categories indicated in (**C**). (**D**) Representative phase and immunofluorescence images show that stromal areas in patient samples with a tumor that has disseminated to a lymph node (T3N1) show areas with high levels of phosphorylated β-catenin, not present in tumors without stromal invasion to lymph node (T2N0) as predicted in A; Quantification shown in (**E**). For C, E; *n* = 10 locations, from 3 sections of the indicated patient samples; Scale bar = 100 µm. Error bars: s.e.m.; *p*-values: *: *p* < 0.05, **: *p* < 0.01; ****: *p* < 0.0001.

To identify the transcription factors which might explain the large differences in gene expression of fibroblast clusters, we calculated the upstream regulators using Ingenuity Pathway Analysis (Figure 2A). Selecting four significant upstream transcription factors (TF) as regulators for each cluster, we calculated their activation across all clusters. Activated TF analysis revealed both expected and surprising findings. TP53, which encodes the tumor suppressor p53 is predicted to be activated in cluster 0, 2, and 4, suggesting quiescent fibroblasts. Cluster 2 constitutes nearly all of the fibroblasts in normal patients, while cluster 4 appears primarily in stage IIB, when definitive stromal trespassing has occurred. Surprisingly, fibroblasts from normal patients were also predicted to have high activation of STAT3 and STAT5B signaling, as well as NFKB1a activation, indicating an inflammatory state. We find low but significant activation of HIF1A in cluster 0, indicating that onset of hypoxia starts within primary stage to affect surrounding fibroblasts. HIF1A stays highly activated through the rest of the cancer in cluster 3, 5, and 7. Stage IIA, when the tumor has grown large but not yet metastasized to the lymph largely contains cluster 5 fibroblasts, which have high HIF1A, as well as ARNT that encodes HIF-1β subunit of the HIF complex. To confirm the surprising finding, we obtained tissue samples from PDAC patients at tumor stages corresponding to the stage IIA and IIB, denoted in the standard TNM score. Immunohistochemistry using Apotome-based sectional illumination showed that in the patient PDAC sample with large tumor mass but no lymph metastasis, many stromal areas had high HIF-1a staining. There were regions with very high, as well as moderate HIF-1a levels, both significantly higher compared to a sample from a patient with early lymph metastasis (T3N1) (Figure 2B,C). As the tumor mass grows larger, tissue hypoxia can set in, resulting in high HIF-1a activation, which may be relieved as the tumor disseminates, possibly due to correlated vascularization, or access to oxygen in smaller disseminated nodes. How much does HIF-1 play a role in regulating the transition to a metastatic state is difficult to predict from these data, but it is certainly notable that the main fibroblast subpopulation in stage IIA have high HIF-1 activation. 

In addition, fibroblasts in cluster 0 show activation of serum response factor (SRF), as well as myocardin-related transcription factor A (MRTFA), both key players in regulating cellular contractility. MRTF-A is well known to control the myofibroblast differentiation of stromal cells [30], and it is notable that cluster 0 is mostly activated in IIB, when stromal trespass has occurred. Various reports have recently indicated that highly contractile CAFs can play a crucial role in regulating cancer dissemination, either by direct interaction with epithelial-type cancer cells [31] or by generating distal mechanical force fields [32]. Other clusters marking the dramatic transition in fibroblast constitution across stages are cluster 1, 3, 5, and 6, which are present in high proportions in stage IB, but re-emerge strongly in stage IIB after disappearing in the transient stage IIA. Cluster 1 showed a high activation for SMARCA4, which encodes a protein BRG1 involved in many SWI/SNF protein complexes, involved in chromatin remodeling, and DNA repair processes [33]. Cluster 3, which also shows high HIF-1A activation, is also predicted to have high CREB1 activation, which is a cyclic AMP binding TF, as well as β-catenin (CTNNB1). Both are central regulators of Wnt signaling and indicate a high fibrotic activity [34,35,36]. These TFs are also activated in cluster 4, with additional activation of SNAI1 and SP1. SNAI1 encodes Snail-1 which regulates the mesenchymal transition of cancer, and also pro-metastatic CAFs [37,38], while SP1 is critical for the production of various ECM proteins [39]. Overall, it appears that the transition from stage IIA to stage IIB is therefore characterized by diverse phenotypes, including increased contractility, ECM production, fibrotic response which are distributed across many fibroblast subclasses, together orchestrating a pro-metastatic response. Finally, this transition also involves the emergence of new subclasses: cluster 7 and 8, which appear to be mostly pro-inflammatory with high predicted activation of STAT3, CREB1, and RELA, as well as high myc activation. To confirm stage-wise activation of predicted TFs in patient stroma, we tested TF activation using immunohistochemistry in patients before and after lymph metastasis (N0 and N1, respectively). Because cluster 5 constituted the bulk fibroblast population in stage IIA (T2/3N0), which was completely replaced with many other clusters, including cluster 4 after lymph metastasis (T2/3 N1), we obtained patient samples representing either stage for phospho b-Catenin levels, which was predicted to be largely present in cluster 4, but not in cluster 5. Indeed, stage IIB (N1) sample had significantly higher levels of phosphorylated b-Catenin levels in the stromal region characterized by sparse nuclei, compared to the non-metastasized sample from stage IIA (N0) (Figure 2D,E). 

Overall, a fibroblast-centric analysis of PDAC progression showed large changes in stromal reaction to cancer as it progresses towards metastasis. Cancer-associated fibroblasts were very different from fibroblasts from a normal pancreas, the latter largely encoding genes associated with physiological functions in the pancreas, indicating either a dramatic shift in the fibroblast state, or more likely a different origin of CAFs. As the tumor grew, the heterogeneity of the fibroblasts actually decreased dramatically (from IB to IIA), and this shift was associated with the emergence of a homogenous class of fibroblasts marked by matrix remodeling phenotype. It would appear that the growth of the tumor resulted in the activation of fibroblasts, resulting in activation of wound healing response, activating canonical pathways associated with matrix production and remodeling. Surprisingly, these fibroblasts were nearly completely replaced by a highly heterogeneous population as the cancer cells trespassed the stroma to reach proximal lymph nodes (Stage IIB). Matrix remodeling phenotype was replaced by either overactivated fibroblasts (cluster 0, 7) or those inhibiting matrix remodeling (cluster 4), vasoregulation (cluster 1), and antioxidant activity (cluster 6). 

### 3.2. Fibroblasts in Succeeding Metastatic Stages Exhibit Change from Physiological to Pathological Activation

**Figure 3:**
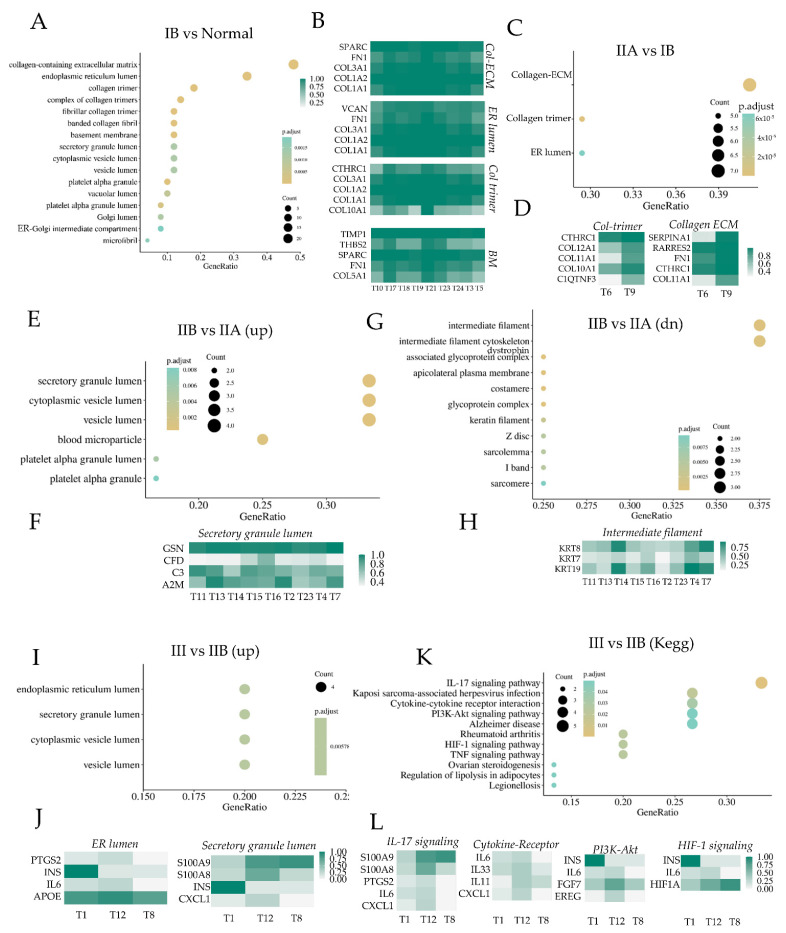
Pancreatic ductal cell carcinoma progression is accompanied by changes in fibroblast phenotypes across cancer stages. (**A**) Gene ontology (GO) enrichment score in all fibroblasts at stage IB differentially compared to fibroblasts from a normal pancreas; top differentially expressed genes in chosen GOs shown for fibroblasts from all patients at stage IB shown in (**B**). (**C**) GO enrichment score in all fibroblasts at stage IIA differentially compared with stage IB; top differentially expressed genes in chosen GOs shown in (**D**). (**E**–**H**) GO enrichment score for upregulated ontologies in fibroblasts at stage IIB compared to stage IB (**E**), and for downregulated ontologies (**G**); top differentially expressed genes for chosen upregulated or downregulated GOs in IIB vs. IIA shown in (**F**,**H**), respectively. (**I**–**L**) GO (**I**) and Kegg (**K**) activation score for upregulated gene sets in fibroblasts at stage III compared to IIB; top differentially expressed genes for samples obtained from patients at stage III shown for chosen GOs (**J**) and Kegg pathways (**L**). For all the above differential gene expression analyses, the criteria used were: *p*-value ≤ 0.05, log2fc ≥ 1 for upregulated gene sets and log2fc ≤ −1 for downregulated gene sets.

Having identified that PDAC cancers, which are remarkable for their high stromal content and heterogeneity, show large changes in fibroblast heterogeneity in succeeding stages of early metastasis, we directly analyzed the gene expression changes in fibroblasts in each succeeding stage. Gene ontology (GO) and KEGG signaling pathway enrichment analysis of fibroblasts showed the activation of many ontologies associated with matricellular protein assembly (Figure 3A and Figure S5). Gene expression analysis across all cancer samples for the ontologies showed high expression of genes encoding fibronectin (FN1) and collagen chains, particularly collagen-1A and collagen-3A, associated with fibrosis and connective tissue, as well as those for collagen 5A and SPARC which encodes a cysteine-rich acidic matrix-associated protein (Figure 3B and Figure S5). Positive enrichment of GOs was limited in fibroblasts as cancer moved from a minor detectable lesion to a larger tumor non-metastatic mass (Stage IIA), with GO enrichment moving further in the direction of increased matricellular protein production (Figure 3C and Figure S6). Interestingly, stage IIB resulted in an increased expression of genes encoding constituents of collagen X (COL10A1), collagen X1 (COL11A1), and collagen XII (COL12A1) isoforms, which have been reported to be prognostic markers of pancreatic cancer metastasis [40,41] (Figure 3D and Figure S6). In addition, we found another GO, collagen-associated ECM with high expression of RARRES2, encoding a tumor suppressor retinoic acid receptor responder also called chimerin [42] which phosphorylates β-catenin reducing fibrotic activity [43], CTHRC1 encoding a secreted Wnt modulator called collagen triple helix repeat containing 1, and a regulator of the healing scar process in myocardial infarction [44], and SERPINA1 encoding anti-trypsin peptidase antagonist. Overall, these genes indicate that stage IIA progression is associated with the emergence of fibroblasts with an anti-tumor growth and dissemination effect. 

The most dramatic effect in fibroblast heterogeneity was observed in the transition of PDAC cancers from IIA to IIB, which is characterized by the first detected stromal trespassing of cancer cells to proximal lymph nodes (Figure 3E and Figure S7). GO enrichment analysis showed that transition to IIB resulted in increased activation of ontologies associated with secretory machinery and downregulation of intermediate filaments and epithelial-like gene sets within fibroblasts. Gene expression analysis showed increased expression of GSN encoding a cytoskeletal protein, Gelsolin, a downstream effector of Rac increasing fibroblast motility and actin dynamics [45], and complement factor D (encoded by CFD), which increases MMP1 production in various contexts and is described as a strong marker for inflammatory CAFs (iCAFs) in PDACs [46] (Figure 3F). Additionally, an increased expression of other cytokines was also revealed, including IL6, CXCL1, as well as INS which encodes insulin, and S100 family of calcium binding proteins S100A8 and S100A9 (Figure S7). S100A8 and 9 are also referred to as alarmins, which are inflammatory cytokines released in response to cellular triggers and induce the release of classical inflammatory cytokines [47], as well as mediate cytoskeletal rearrangement and arachidonic acid metabolism [48]. Both alarmins are constitutively expressed in neutrophils, and their expression in fibroblasts along with IL6, CXCL1, and INS indicate the activation of a pro-inflammatory state. In contrast, the progression to stage IIB associated with stromal trespassing was accompanied with a decreased expression of keratins, KRT7/8/9 (Figure 3G,H and Figure S7). 

As the cancer becomes more metastatic spreading to a larger number of lymph nodes (stage III), we found further upregulation of gene sets associated with the secretory cellular machinery (Figure 3I and Figure S8), indicating an increase in the inflammatory phenotype among fibroblasts. Top genes in each gene set showed high expression of genes encoding interleukin-6 and cyclooxygenase-2 encoded by PTGS2 gene (Figure 3J). In addition, there was a continued increase in expression from stage IIA to IIB, and then to III of pro-inflammatory S100A8 and S100A9, encoding for alarmin isoforms that exist as a heterodimer. KEGG enrichment analysis also showed an increase in pro-inflammatory phenotype among fibroblasts with increased enrichment in IL-17 signaling, HIF-1 signaling, and cytokine-receptor signaling (Figure 3K). Top genes in these gene sets revealed higher expression of IL6, IL11, IL33, CXCL1 encoding inflammatory cytokines, as well as growth factors encoding genes such as INS (encoding insulin), EREG (encoding epiregulin), and FGF7, all of which have been implicated as pro-metastatic factors [49,50] (Figure 3L). Increased expression of HIF-1A, gene encoding hypoxia-inducible factor-1 alpha, the master regulator of oxygen sensing and metabolic regulation in cells, and INS encoding insulin further indicate that fibroblasts assume a pro-metastatic role and are complicit in pancreatic cancer progression [51,52]. Pancreatic fibroblasts have been reported to increase cancer dissemination and motility mediated by IGF1/IGF1R signaling under hypoxia, which is also reported to increase PI3K/Akt activity in fibroblasts as was observed in these data [52]. Interestingly, the continued increase in inflammatory phenotype is also accompanied by a continued decrease in pathways related to myofibroblast activation, marked by genes encoding matricellular proteins, including collagen X and X1 isoforms, as well as COMP and MATN3 (Figure S8). 

### 3.3. Paracrine Signaling by Fibroblasts to Other Cell Type Changes Modestly through the Metastatic Cascade

**Figure 4:**
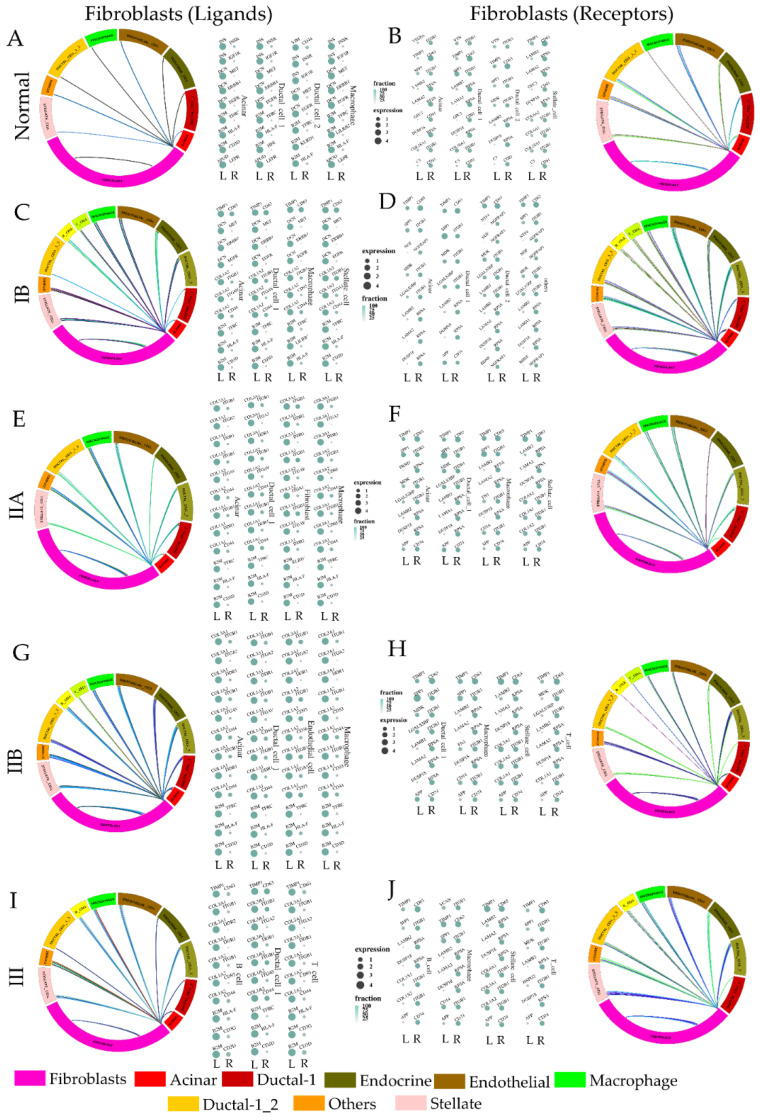
Mapping stage-wise ligand–receptor interactions of fibroblasts with other cell types in pancreatic ductal cell carcinoma. (**A**) Circos plot showing putative interaction of fibroblast-expressed ligand encoding genes in normal pancreas and their expressed cognate receptor genes in other cell types. Each cell type is represented by a different color shown in the figure legend. Interaction represents GO enrichment for type of ligand–receptor interaction between two cell types, each GO shown in different color, width representing strength of interaction. Additionally, the top 4 ligand-expressing genes in fibroblasts are shown with the expression of top 3 genes encoding potential cognate receptors in other cell types. The size of the bubble represents ligand expression. (**B**) Circos and bubble plots showing genes encoding receptors in fibroblasts from a normal pancreas and their putative ligand expression in other cell types; color scheme and other parameters similar to A. Ligand–receptor interactions between fibroblasts and other cell types with fibroblast-expressed ligands for (**C**) stage IB, (**E**) stage IIA, (**G**) stage IIB, and (**I**) stage III, and fibroblast-expressed receptors for (**D**) stage IB, (**F**) stage IIA, (**H**) stage IIB, and (**J**) stage III. For fibroblast-expressed ligands (**A**,**C**,**E**,**G**,**I**), the cut off used was: expression(ligand) ≥ 0.5, fraction of cells expressing ligand ≥ 5%, and ratio of genes in a given GO term for circos plot ≥ 9%. For fibroblast-expressed receptors (**B**,**D**,**F**,**H**,**J**), the cut off used was: expression(receptor) ≥ 0.5, fraction of cells expressing receptor ≥ 10%, and ratio of genes in a given GO term for circos plot ≥ 7%.

We identified the genes encoding ligands expressed in fibroblast cells at every stage of cancer, matched to their cognate receptors in other cell types present at the same stage. In normal pancreatic tissue, most interactions by fibroblasts with other cell types indicated their supporting stromal role (Figure 4A,B and Figure S9). These included the expression of genes such as INS, DCN, and B2M encoding insulin, decorin, and beta-2 microglobulin. Fibroblasts also expressed high levels of CD81 indicating exosome-mediated intercellular communication within the pancreatic milieu [53,54]. Interestingly, all these genes have been reported to be increased in PDAC cancers, suggesting that stromal support of cancer invasion is physiologically present in the pancreatic milieu, and it is the low expression of their cognate receptors, or ligands in the ductal cells which limits these pro-metastatic signaling, and primarily prevents tumor growth and dissemination. At stage I, where the tumor is present, the largest difference in fibroblast signaling to neighboring cells involves increased expression of TIMP1, and collagen isoforms suggesting an early fibroblast activation (Figure 4C,D and Figure S10). As the cancer grows larger in stage IIA, without observable lymph node metastasis, the ligand–receptor interaction with fibroblasts changes significantly (Figure 4E,F and Figure S11). Fibroblasts express high amounts of matricellular ligands, including those encoding collagen 1 and collagen 3. This is also accompanied by the high expression of CD74, which is a strong marker for pancreatic carcinomas with poor prognosis [55]. CD74 is a chaperone which associates with MHC II, regulates antigen presentation, and strongly indicates perineural invasion [56]. Surprisingly, top ligand encoding genes did not change dramatically with stage IIB and III, but the expression of cognate receptors in other cell types showed changes (Figure 4G–J, Figures S12 and S13). RPSA, which encodes ribosomal protein SA has acquired the role of a high affinity laminin receptor in vertebrates, also linked to pro-metastatic behavior remained highly expressed in fibroblasts, but interestingly T-cells show an increase in collagen levels in IIA, which reverts back to laminin isoforms in stages IIB and III [57,58]. Additionally, TIMP1 expression in fibroblasts again emerges in stage III when cancer has become decidedly metastatic. Overall, the most dramatic changes in fibroblast-expressed ligands occurred in the initial stages of cancer progression, probably priming the pre-metastatic transition. 

### 3.4. Transition to Early Metastasis in PDAC Cancer Is Associated with a Dramatic Reduction in Genes Evolved to Resist Invasion

**Figure 5:**
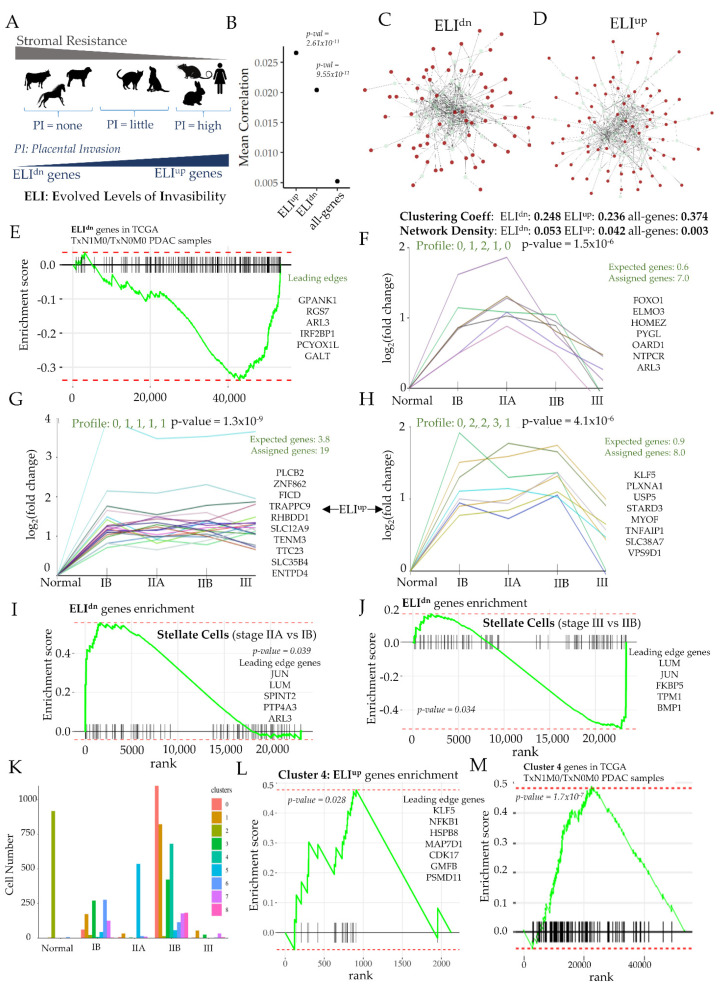
Transition from pre-metastatic to metastatic cancer is accompanied by systemic downregulation of evolved stromal resistance and emergence of fibroblast subclass with increased vulnerability to invasion. (**A**) Schematic showing ELI (Evolved Levels of Invasibility) framework highlighting stromal genes which increase expression correlated to increased stromal invasibility (ELI^up^) or decreased stromal invasibility (ELI^dn^) to placental or cancer invasion. ELI^up^ genes are predicted to be pro-invasive and ELI^dn^ genes are predicted to be resistive. (**B**) Gene–gene correlation analysis in PDAC fibroblasts for all genes, top 100 ELI^up^ genes, and top 100 ELI^dn^ genes. (**C**,**D**) Connected subnetworks of ELI^up^ and ELI^dn^ genes in PDAC fibroblasts across all stages created upon Reactome validated protein–protein interaction network; red nodes refer to top 100 ELI^up^ (**C**) or ELI^dn^ (**D**) gene sets, while gray nodes refer to linker genes extracted in Reactome to create a connected subnetwork. (**E**) GSEA enrichment analysis showing negative enrichment of resistive ELI^dn^ genes among the differential gene set between N1 (lymph metastasis) and N0 (no lymph metastasis) PDAC cancers from TCGA database; *n*(N1) = 312; *n*(N0) = 116 patients; Additionally, leading edge genes are shown. (**F**) STEM analysis revealed a significant pattern of gene expression changes in fibroblasts across different stages of PDAC cancer within the top 100 ELI^dn^ genes; pattern profile shown in green. (**G**,**H**) STEM analysis revealed significant patterns of the top 100 ELI^up^ genes whose expression changes with stages of cancer are significant. (**I**,**J**) Enrichment of ELIdn set in stellate cells among the differential genes between stage IIA and IB (**I**), and between stages III and IIB (**J**); leading edge genes and *p*-values are shown in panel. (K-M) Stromal trespass of cancer from primary lesion to proximal lymph nodes is accompanied by emergence of a fibroblast subclass with ELI predicted pro-invasive phenotype; (**K**) Distribution of fibroblast clusters determined in Figure 1A across different stages of PDAC cancer; (**L**) GSEA enrichment of ELI^up^ genes within the cluster 4 upregulated gene set. (**M**) GSEA enrichment of cluster 4 upregulated genes among the differential gene set between N1 (lymph metastasis) and N0 (no lymph metastasis) samples from a larger cohort of bulk RNAseq data from TCGA.

Based on comparative transcriptomics of eutherian species with vastly different stromal response to invasion, we have identified genes that have changed expression correlated to the extent of resistance (or invasibility) they offer [20]. We have also experimentally validated that when genes with reduced expression in resistive stroma (e.g., bovines and equines) are silenced in human fibroblasts, their invasibility to epithelial invasion is reduced [59]. Based on this framework, termed Evolved Levels of Invasibility (ELI), ELI^dn^ genes encode gene products which increase stromal resistance to invasion, while ELI^up^ genes encode gene products which increase stromal invasibility, within the context of the evolved changes in stromal invasibility across mammals (Figure 5A). Transcriptomic analysis of fibroblasts in PDAC had revealed large changes in fibroblasts at different stages of cancer. To test if ELI genes play a significant role in fibroblasts of PDAC, we computed gene–gene correlation for all genes, as well as ELI gene sets taking advantage of the large number of samples available in scRNAseq data, with each cell being a new instance. We surprisingly found that ELI gene sets were significantly more correlated than the rest of the genes within fibroblasts, indicating that ELI-related genes may be systematically important in fibroblast transitions (Figure 5B). Using an experimentally validated protein–protein interaction (PPi) network from Reactome knowledgebase [60], we measured the connectivity of ELI genes within the whole PPi interaction network. Interestingly, selecting top 100 ELI^dn^ and ELI^up^ genes, along with linker genes, showed lower clustering coefficients than all genes, but very high network density, suggesting a highly hub-spoke-like architecture (Figure 5C,D). We found that with the addition of a relatively few linker genes, both top 100 ELI^up^ and ELI^dn^ could be extracted as densely connected subnetworks (Figure 5C,D), supporting the hypothesis that within pancreatic cancer fibroblasts, evolved stromal integrity or the lack thereof is systematically implicated. 

We therefore asked whether the expression of these ELI genes change with the progression of cancer. ELI genes are specifically predicted to relate to evolved stromal resistance, and therefore are expected to be mostly in play at the stage when cancer starts disseminating and trespassing the stromal compartment to the lymph nodes. We therefore collected gene expression data from PDAC patients listed in the TCGA (The Cancer Genome Atlas) database [61]. We specifically concentrated on patients with detected lymph node metastasis (TxN1M0) and those without any lymph node metastasis (TxN0M0), which correlate with stage IIA and IIB, respectively. To recall, the most dramatic changes in fibroblasts were also obtained in the transition from IIA to IIB. GSEA analysis showed a remarkable negative enrichment of the top 100 ELI^dn^ genes in samples from patients transitioning from non-metastatic to early lymph node metastatic cancers (Figure 5E). Most genes in this small subset were marked as leading edge genes (Figure S14), including many potential tumor suppressors or anti-metastatic genes (Figure 5E). These included RGS7 encoding regulator of G-Protein signaling 7 important in cellular contraction by regulating calcium signaling through association with the Gb5 subunit of GPCRs [62], MDFI encoding a myodin inhibitor, DUSP8, a potent dual specificity phosphatase which could inactivate many proliferative pathways including MAP kinase superfamily including SAPK/JNK and p38 [63], as well as tumor suppressor NPRL2 [64]. Interestingly, DUSP8 is induced by insulin or NGF, both ligands presented by multiple cell types in the pancreas as indicated by ligand–receptor expression maps (Figure 4). Other anti-invasive stromal ELI^dn^ genes downregulated from stage IIA to IIB transition included ELMO3 encoding engulfment and cell motility-3, suppression of which within cancer cells is known to suppress cancer invasion [65], but its effect on the resistive stromal cells may be opposite, cancer-related NTPCR encoding nucleoside triphosphatase. Additionally, WDR17 and WDR83 were remarkable, which encode members of the WD-40 family involved in stabilization of HIF-1α, and also interaction with Erk pathways. Interestingly, many mitochondria-associated ELI^dn^ genes were downregulated in fibroblasts with metastatic transition of cancer, including COX10, MSTO1, CPT2, and MDH1B. 

### 3.5. Stromal Trespass of PDAC Is Associated with Emergence of a Fibroblast Subtype with Evolved Stromal Invasibility Gene Expression

We then tested whether anti-invasive ELI^dn^ and potentially pro-invasive ELI^up^ genes show any significant pattern in stage-wise stromal expression. We conducted a Short Time-series Expression Mining (STEM) analysis [27] to identify significant patterns in either gene set within fibroblasts along PDAC cancer stages. For anti-invasive ELI^dn^ genes which are more highly expressed in cow and horse stroma, we found a significant pattern of genes which continued to increase expression till stage IIA, and then decreased expression as the cancer trespassed stromal compartment (Figure 5F). In contrast, the significant patterns for potentially pro-invasive ELI^up^ genes, more expressed in human and rodent stroma, showed two significant patterns with one showing a sustained increase in expression in response to tumor. These genes were increased in stage IB itself and remained so through all other stages, and may likely promote stromal invasibility to cancer dissemination (Figure 5G). Interestingly, a smaller set of genes also showed another a significant pattern, wherein genes increased expression with tumorigenesis and increased further upon lymph node metastasis (stage IIB), and then reducing to the level of normal stroma in stage III (Figure 5H). Interestingly, and in the pattern of fibroblasts, pancreatic stellate cells, which are also considered as chief effectors of pancreatic fibrosis [66,67], also show a strong upregulation of pro-resistive ELI^dn^ genes as the tumor becomes larger (stage IB to IIA) (Figure 5I). As the cancer metastasizes, these genes then show a reversal, with pro-resistive ELI^dn^ genes negatively enriched in stage III vs. IIB (Figure 5J). Although we concentrate here on the fibroblasts, it is notable that stellate cells show a similar trend of an initial increase in ELI-predicted pro-resistive gene expression, and its eventual reversal is accompanied by metastatic transition. It is also noteworthy that we did not find significant gene enrichment of ELI genes (both up and down) in stellate cells in stage IIB vs. IIA, indicating that the decrease in resistive genes in stellate cells follow the direction of fibroblasts which already exhibit reduced ELI^dn^ expression at initial lymph metastasis in stage IIB. Furthermore, it is notable that ELI^dn^ signature in TCGA PAAD cancers (95 patients) was strongly correlated with immunosuppressive signature in pancreatic cancers [68] (Figure S15A). Similarly, we noticed that genes identified in several studies to confer sensitivity to gemcitabine [69,70], an important chemotherapeutic with positive prognosis in PDAC cancer, were positively correlated to ELI^up^ signature (Figure S15B). A similarly significant trend was found for cluster 5 fibroblast genes in the TCGA database, both for well-known immunosuppressive genes (Figure S15C), and genes when silenced have been found to cause drug resistance (Figure S15D). Similarly, we found that both ELI^up^ and ELI^dn^ signatures were correlated with many gene mutations present in pancreatic cancer, as analyzed by systemic analysis of the TCGA pancreatic cancer patient data (Figure S15E,F). However, we note that because these data are based on bulk gene–signature correlation in patient data, our conclusion cannot make specific or mechanistic claims about the role of stromal gene expression in conferring both immunosuppression, chemoresistance, or tumor mutations. 

We therefore finally tested if this change in fibroblast diversity across cancer stages showed the enrichment of pro-invasive fibroblasts. As noted earlier, the transition from stage I to stage IIA is characterized by a homogenization of fibroblasts, mostly grouped in cluster 5 in our initial t-sne analysis. As cancer breaches its boundary to trespass through stromal compartment to the lymph nodes, many other clusters emerge (0, 1, 3, 4), and to a lesser extent cluster 6 and 7 (Figure 5K). We asked if any of these newly emerging clusters showed any significant enrichment for pro-invasable ELI^up^ genes. We only found one cluster (cluster 4) with significantly high enrichment of ELI^up^ genes, suggesting that cluster 4 subtype of fibroblasts may potentially be pro-invasive (Figure 5L). Interestingly, all the leading edge genes indicated a pro-oncogenic or pro-metastatic phenotype, including KLF5, Kruppel-like Factor 5 associated with poor survival in PDACs [71,72], NFKB1, nuclear factor kappa-light chain enhancer of activated B cells which is oncogenic and metastatic enhancers in PDAC cancers [73], heat shock protein binding 8 (HSPB8) which is oncogenic [74], MAP7D1 with unfavorable prognosis in liver and renal cancer [75], GMFB which encodes glia maturation factor beta associated with multiple types of cancers [76], as well as PSMD11, a biomarker for PDAC cancer [75]. This suggested that the emergence of cluster 4, with high enrichment of pro-invasive ELI^up^ genes which are all reported to be pro-metastatic, may play a role in the transition of cancer from a non-metastatic to a metastatic state (Figure S16). Finally, we tested if cluster 4 fibroblasts were indicative of stromal trespass and transition to metastasis in a larger cohort of PDAC cancer data. GSEA analysis of PDAC patient data from lymph node metastasis (N =1 in TNM score) vs. those without any lymph metastasis (N = 0 in TNM score) showed a high enrichment of cluster-4-related genes (Figure 5M). These data strongly indicate that metastatic transition of PDAC cancer is accompanied by the (i) downregulation of resistive ELI^dn^ stromal genes and (ii) emergence of a subclass of fibroblasts with pro-invasable ELI^up^ signature, both data being confirmed in the larger patient cohort data from TCGA.

### 3.6. Genes Characterizing PDAC Fibroblast Subtypes Partly Confer Pro-Invasable and Pro-Resistive Stromal Phenotype

**Figure 6:**
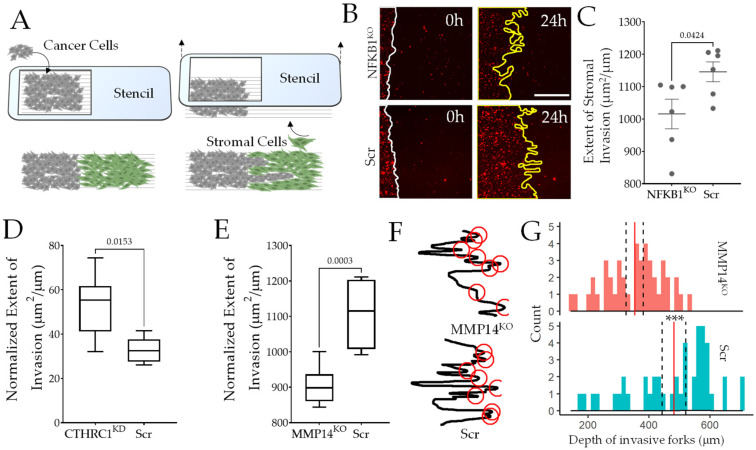
Experimental validation of causality of genes identified in pro-invasable and pro-resistive stromal clusters to regulate stromal resistance to cancer invasion. (**A**) Schematic showing setup to measure stromal invasibility, ANSIA (Accelerated Nanopatterned Stromal Invasion Assay); fluorescently labeled cancer cells are patterned with stromal fibroblasts to create a collective interface between the two orthogonal to the underlying anisotropic nanopatterned fiber direction; cancer invasion is measured for 24 h using live cell fluorescent microscopy. Here, invading cancer cells are the constant, while the invaded fibroblasts are perturbed with siRNA- or CRISPR/Cas9-mediated gene silencing. (**B**) Representative images of cancer cells invading BJ fibroblasts with or without CRISPR/Cas9-based gene knockout for NFKB1, a key leading gene in pro-invasable cluster 5, with quantified extent of cancer invasion shown in (**C**). (**D**) ANSIA-based quantification of stromal invasion with a key gene characterizing pro-resistive cluster 5, CTHRC1 silenced in fibroblasts. (**E**) ANSIA-based quantification of stromal invasion with a key gene characterizing pro-invasable cluster 4, MMP14 silenced in fibroblasts. (**F**) Representative invasive front interface of cancer cells into the monolayer of control fibroblasts, and silenced for MMP14; Automated peak identification denoted by red circles; (**G**) Distribution of the length of invasion in either stromal monolayer by identified invasive forks shows that MMP14 gene silencing reduces deep collective invasion. In (**D**,**E**), *n* = 6 locations; in (**G**), *p*-value = 9.948 × 10^−7^, denoted as ***.

Stage-wise analysis of heterogeneous fibroblast clusters in PDAC cancer revealed a dynamic stromal environment that changed from resistive to pro-invasive as the tumor becomes metastatic. Based on our theory of Evolved Levels of Invasability, as well as combined gene set enrichment analysis on larger PDAC patient data from TCGA, there was a strong indication that as a tumor becomes larger, fibroblasts are activated with a strong resistive signature, while as it disseminates into proximal lymph node, the stromal trespassing is associated with a marked disappearance of resistive fibroblasts (cluster 5) and emergence of a distinct category of pro-invasive fibroblast subtype (cluster 4). We therefore sought to experimentally test if genes characterizing these clusters indeed confer the said phenotype to fibroblasts in general. We used ANSIA, a method we have previously described, abbreviated for Accelerated Nanopatterned Stromal Invasion Assay, which allows the observation of cancer invasion dynamics into the stromal compartment in an accelerated time frame, owing to the orthogonal arrangement of collagen fibrils mimicking nanotextured ridges underneath the monolayers. Fibroblasts can be genetically perturbed while keeping the fluorescently labeled invasive cancer cells as the constant to specifically test the role of stromal genes in regulating stromal invasability (or resistance) (Figure 6A). We tested the effect on NFKB1, a key leading gene identified in cluster 4, predicted to promote the invasion of ELI (Figure 5L). CRISPR/Cas9 mediated gene knockout of NFKB1 in BJ fibroblast resulted in a significant reduction in stromal invasion vs. scrambled control, confirming that NFKB1 decreases stromal resistance to invasion (Figure 6B,C),and may contribute to the pro-invasable cluster 4 phenotype present in PDAC cancers with lymph metastasis. 

To experimentally confirm the causality of genes characterizing cluster 5 and cluster 4, predicted to be pro-resistive and pro-invasable by our ELI framework, their emergence correlating with the onset of early metastasis, we tested a candidate gene from the key genes characterizing either cluster (Figure 1I, Figures S2–S4). We chose CTHRC1, gene encoding Collagen Triple Helix Repeat Containing 1, involved in myofibrosis and regulator of the healing scar process [44] present in cluster 5. ANSIA-based stromal invasion analysis showed that silencing of the CTHRC1 in fibroblasts markedly increased cancer invasion, confirming its role as a contributor to stromal resistance characterizing cluster 5, which predominates the stromal fibroblast population in stage IIA, as cancer grows but is yet to disseminate. In contrast, we found that silencing a key gene in cluster 4, MMP14 reduces invasability in fibroblasts. MMP14 is a clinically relevant target to image and treat several metastatic cancers [77,78]. Finer spatial analysis of the cancer–fibroblast interface and identification of the tip of invasive forks which directionally aligned parallel to the underlying nanofibers revealed that when MMP14 was silenced in stroma, the depth of invasive forks was markedly reduced suggesting that MMP14 contributed to fibroblast invasability, probably by assisting stromal matrix degradation (Figure 6F,G). These data confirmed that genes characterizing distinct fibroblast subpopulations dominating the pre-metastatic and early metastatic stages regulate stromal invasability, highlighting the causative and regulatory role of stromal fibroblasts in mediating the transition of pancreatic cancer towards a metastatic disease. 

## 4. Conclusions

It is now well appreciated that although tumorigenesis may be largely an autonomous function of cancer cells, its progression to a metastatic disease depends on factors beyond the biology of cancer cells themselves, involving many other stromal components [1,79]. Among these, cancer-associated fibroblasts (CAFs) [80,81] as well as macrophages [82,83,84] have been extensively studied, and many mechanisms are now known through which these cell types cooperate with cancer cells towards metastasis. 

Although the changes in fibroblast activation as cancer progresses have been detailed, there is still a lack of coherent understanding of how fibroblasts may orchestrate or assist in the transition of cancer from a non-malignant to a malignant phenotype. Do they actively pull and disseminate cancer cells from the boundary of their primary location, or do they merely allow cancer cells to pass through the stromal compartment by increasing their invasibility? What mechanism induces fibroblasts to change these phenotypes, since these are clearly not inherent properties of normal fibroblasts? Additionally, are all changes in the fibroblasts also cancer induced, wherein cancer is still the active player, recruiting fibroblasts to its own assistance? These are crucial questions, because even if we understand the mechanisms which render CAFs to be assistive in metastasis, the mechanism is still positioned from the perspective of the presumed active player, the cancer cell. What selective advantage does a fibroblast have in allowing cancers to invade into the stromal compartment? If cancer metastasis is a pathological incorporation of other physiological mechanisms through which fibroblasts assist in cancer invasion, could we pose our question from that physiological context? 

The evolutionary framework, Evolved Levels of Invasibility (ELI), based on a remarkable and large change in placental invasion across mammals identifies the evolved changes in stromal resistance (or invasibility) as a phenotype [7]. This framework centers fibroblasts as key players in regulating stromal invasibility, and not as passive or secondary partners to the invading cancer cells (or trophoblasts). We have previously shown that changes in the ELI genes are significantly linked to cancer metastasis and survival, particularly in melanoma [10]. In particular we found that the ELI^dn^ genes which are higher in cows, and potentially impart stromal resistance to its fibroblasts, are reduced in human melanoma. These resistive genes, when lost, correlate with increased cancer metastasis and reduced survival.

Here, we have attempted to explore the changes in the fibroblast landscape as cancer progresses from an initial tumor mass through the metastatic cascade. We chose pancreatic cancer as an appropriate model owing to its particularly high stromal content, as well as its lethality. Overall, we found the classical fibroblast response to cancer [81], with nuances and new details. Fibroblasts from normal patients largely acted as stromal support to the parenchymal cells of the pancreas, the acinar and the ductal cells, but at stage I, when the tumor is small, the response shifted dramatically to being that of activated fibroblasts responding to the primary lesion as a wound. In stage IIA, when the tumors have become larger but have still not disseminated to the lymph nodes, the fibroblasts surprisingly presented a highly homogenous front. This was characterized by a large increase in transcripts encoding matricellular proteins and significant activation of HIF-1a signaling even though HIF-1 accumulation is considered to be mostly present within the tumor core. However, it is the transition of the cancer from a pre-metastatic to an early metastatic stage where the most dramatic shift occurred within the fibroblasts. Patient samples representing these stages showed higher HIF-1a levels in stage IIA, with much reduced levels in stage IIB. Comparing samples from stage IIA and stage IIB, characterized by proximal lymph metastasis, we found a pronounced increase in fibroblast heterogeneity, showing the emergence of new subclasses of fibroblasts. These subclasses constituted pro-inflammatory fibroblasts, as well as crucially, a subclass with high expression of contractility genes, genes encoding collagen X/XI/XII and MMPs, as well as predicted the activation of β-catenin, which we also confirmed in patient tumor samples representing stage IIA and IIB. We also found that the inflammatory signature increased overall after lymph node metastasis had occurred. 

Because scRNAseq allows a cell-type resolved analysis of these changes, we asked if the ELI signature is present specifically in fibroblasts at different stages of cancer, as well as in different fibroblast clusters. Using gene–gene correlation data within fibroblasts, which could each serve as an individual sample, we found that the ELI genes were significantly much more correlated to each other than other genes, indicating a coherent role of ELI genes in fibroblasts. Crucially, we found that resistive ELI^dn^ genes (which are expressed higher in bovine and equine stroma) were downregulated in fibroblasts as PDAC cancer transitioned from a pre-metastatic to an early metastatic stage. Notably, this trend was also observed for stellate cells, which are key effectors of tissue fibrosis in the pancreas, although the downregulation of ELI^dn^ genes was observed later (in stage III) in stellate cells. Furthermore, in a larger cohort of PDAC patient data from TCGA, we found that ELI^dn^ genes were reduced in patient samples with early lymph metastasis vs. those with no lymph metastasis, a finding similar to melanoma [10]. Again, this finding indicated a pattern emerging in fibroblasts, wherein loss of gene expression of ELI^dn^ genes is accompanied by the onset of metastasis. Other analyses further confirmed these patterns. In a somewhat contrasting light, we also found that most top ELI^up^ genes were upregulated with the onset of tumor itself, indicating that the stromal preparation for invasibility is laid earlier than when metastatic events occur, while the resistive defenses are reduced as the cancer cells trespasses the stromal compartment to reach proximal lymph nodes. Finally, an interesting finding with a significant implication for potential therapeutics targeted at stroma is the observation that a particular fibroblast subclass (cluster 4 in the current analysis) is both enriched for ELI^up^ genes, and in patients as cancer metastasizes to a lymph. This subclass is characterized by Wnt activation, cyclic AMP driven transcription, and SP1 activation, and emerges in fibroblasts at stage IIB as the cancer trespasses the stroma. 

Most of our understanding of the mechanisms driving cancer metastasis is centered on cancer cells as the primary agent, with the stromal environment considered as a passive barrier. Our framework suggests that stromal fibroblasts may be active regulators of the process of cancer metastasis itself. Resolved stage-wise analysis of fibroblasts in PDAC cancer showed a remarkable dynamic, but the most crucial for regulating cancer metastasis was an initial homogenous fibroblast reaction to contain the tumor, which collapses in cases where metastasis has set in. Two distinct fibroblast clusters, cluster 5 and cluster 4 in the current analysis, were present, respectively, in pre-metastatic stage IIA and stage IIB wherein lymph metastasis has occurred, and were enriched in pro-resistive ELI^dn^ genes and pro-invasive ELI^up^ genes, respectively. We experimentally tested if genes characterizing these fibroblast clusters indeed confer the said phenotypes to fibroblasts in general. We chose to silence CTHRC1 present in cluster 5 and MMP14 present in cluster 4 in BJ fibroblasts and found that the effect of gene perturbation was consistent with the prediction. Measuring stromal invasion where fibroblasts are active players and can also be perturbed is challenging because the process is slow. We have previously developed an accelerated method to assay stromal invasability as a phenotype using a combination of nanotextured substratum, cell patterning techniques, and live fluorescence microscopy. Using this method, we experimentally determined that genes characterizing fibroblast subtypes indeed were partly contributing to the predicted property related to stromal invasability. 

Overall, the data strongly indicate that the stromal trespassing of cancer cells to spread to proximal lymph nodes away from the primary lesion is orchestrated by a dramatic reduction in the stromal invasibility, achieved by systemic changes in gene expression in fibroblasts for anti- and pro-resistive genes. Our detailed transcriptomic exploration of fibroblasts in pancreatic cancer across early stages of metastasis presents a nuanced understanding of stromal response to cancer, and sets it in the context of the regulation of stromal resistance, or invasibility as a selected and evolved phenotype. Our work highlights that published large omics data could be harnessed to provide a deeper understanding of cancer pathogenesis, center cancer-associated fibroblasts as central players in regulating progression of a tumor into a metastatic disease, and provide a roadmap to target stromal biology to contain early cancer dissemination. Our data confirm that the complexity of stromal response to cancer is really a function of stage-wise emergence of distinct fibroblast clusters, characterized by distinct gene sets which confer initially a predominantly pro-resistive and then a pro-invasive property to the stroma. 

## Data Availability

Original data on scRNAseq study was obtained from Peng et al., 2019. Other raw data was obtained from The Cancer Genome Atlas (TCGA). Fibroblast specific data for this study are available on https://github.com/computing-bioX/PDAC-Associated_Fibroblasts (accessed on 27 March 2022).

## Supplementary Materials

The following supporting information can be downloaded at: https://www.mdpi.com/article/10.3390/cancers14092197/s1, Figure S1: Kegg pathway activation in different fibroblast clusters identified in pooled PDAC data for biological processes. Figure S2: Heatmap showing the top 10 genes as markers for fibroblasts from a normal pancreas. Figure S3: Heatmap showing the top 10 genes as markers for fibroblasts from stage IIA. Figure S4: Heatmap showing the top 10 genes as markers for fibroblasts from fibroblasts in stage III, compared to all other fibroblasts. Figure S5: Sample-wise gene expression in the top activated gene ontology in stage IB vs. normal fibroblasts. Figure S6: Sample-wise gene expression in the top activated gene ontology in fibroblasts in stage IIA vs. IB. Figure S7: Sample-wise gene expression in the top activated gene ontology in fibroblasts in stage IIB vs. IIA. Figure S8: Sample-wise gene expression in the top activated gene ontology in fibroblasts in stage III vs. IIB. Figure S9: Ligand–receptor interaction between fibroblasts and other cell types in a normal pancreas. Figure S10: Ligand–receptor interaction between fibroblasts and other cell types in fibroblasts from stage IB. Figure S11: Ligand–receptor interaction between fibroblasts and other cell types in fibroblasts from stage IIA. Figure S12: Ligand–receptor interaction between fibroblasts and other cell types in fibroblasts from stage IIB. Figure S13: Ligand–receptor interaction between fibroblasts and other cell types in fibroblasts from stage III. Figure S14: Leading edge genes in GSEA analysis showing enrichment of ELIdn genes among differentially expressed gene sets in TCGA PDAC patients with (*N* = 1 in TNM score) or without (*N* = 0 in TNM score) lymph node metastasis. Figure S15: Gene–signature correlation in TCGA PAAD cancer for immunosuppression, chemoresistance, and ELI^dn^ genes. Figure S16: Gene ontologies activated in PDAC fibroblast subclass cluster 4.

## References

[1] Suhail Y., Cain M.P., Vanaja K., Kurywchak P.A., Levchenko A., Kalluri R., Kshitiz (2019). Systems biology of cancer metastasis. Cell Syst..

[2] Padmanaban V., Krol I., Suhail Y., Szczerba B.M., Aceto N., Bader J.S., Ewald A.J. (2019). E-cadherin is required for metastasis in multiple models of breast cancer. Nature.

[3] Kang S.H., Oh S.Y., Lee H.J., Kwon T.G., Kim J.W., Lee S.T., Choi S.Y., Hong S.H. (2021). Cancer-associated fibroblast subgroups showing differential promoting effect on hnscc progression. Cancers.

[4] Mizutani Y., Kobayashi H., Iida T., Asai N., Masamune A., Hara A., Esaki N., Ushida K., Mii S., Shiraki Y. (2019). Meflin-positive cancer-associated fibroblasts inhibit pancreatic carcinogenesis. Cancer Res..

[5] Hwang R.F., Moore T., Arumugam T., Ramachandran V., Amos K.D., Rivera A., Ji B., Evans D.B., Logsdon C.D. (2008). Cancer-associated stromal fibroblasts promote pancreatic tumor progression. Cancer Res..

[6] Gore J., Korc M. (2014). Pancreatic cancer stroma: Friend or foe?. Cancer Cell.

[7] Kshitiz, Afzal J., Maziarz J.D., Hamidzadeh A., Liang C., Erkenbrack E.M., Kim H.N., Haeger J.D., Pfarrer C., Hoang T. (2019). Evolution of placental invasion and cancer metastasis are causally linked. Nat. Ecol. Evol..

[8] Wagner G.P., Kshitiz, Dighe A., Levchenko A. (2021). The coevolution of placentation and cancer. Annu. Rev. Anim. Biosci..

[9] Wagner G.P., Kshitiz, Levchenko A. (2020). Comments on boddy et al. 2020: Available data suggest positive relationship between placental invasion and malignancy. Evol. Med. Public Health.

[10] Suhail Y., Afzal J., Kshitiz (2021). Evolved resistance to placental invasion secondarily confers increased survival in melanoma patients. J. Clin. Med..

[11] Reza K.K., Dey S., Wuethrich A., Jing W., Behren A., Antaw F., Wang Y., Sina A.A., Trau M. (2021). In Situ single cell proteomics reveals circulating tumor cell heterogeneity during treatment. ACS Nano.

[12] Atta L., Fan J. (2021). Computational challenges and opportunities in spatially resolved transcriptomic data analysis. Nat. Commun..

[13] Fan J., Slowikowski K., Zhang F. (2020). Single-cell transcriptomics in cancer: Computational challenges and opportunities. Exp. Mol. Med..

[14] Dong M., Thennavan A., Urrutia E., Li Y., Perou C.M., Zou F., Jiang Y. (2021). Scdc: Bulk gene expression deconvolution by multiple single-cell rna sequencing references. Brief. Bioinform..

[15] Jin H., Liu Z. (2021). A benchmark for rna-seq deconvolution analysis under dynamic testing environments. Genome Biol..

[16] Hosein A.N., Brekken R.A., Maitra A. (2020). Pancreatic cancer stroma: An update on therapeutic targeting strategies. Nat. Rev. Gastroenterol. Hepatol..

[17] Peng J., Sun B.F., Chen C.Y., Zhou J.Y., Chen Y.S., Chen H., Liu L., Huang D., Jiang J., Cui G.S. (2019). Single-cell rna-seq highlights intra-tumoral heterogeneity and malignant progression in pancreatic ductal adenocarcinoma. Cell Res..

[18] Thomas D., Radhakrishnan P. (2019). Tumor-stromal crosstalk in pancreatic cancer and tissue fibrosis. Mol. Cancer.

[19] Rasheed Z.A., Matsui W., Maitra A., Grippo P.J., Munshi H.G. (2012). Pathology of pancreatic stroma in PDAC. Pancreatic Cancer and Tumor Microenvironment.

[20] Suhail Y., Maziarz J.D., Novin A., Dighe A., Afzal J., Wagner G., Kshitiz (2022). Tracing the cis-regulatory changes underlying the endometrial control of placental invasion. Proc. Natl. Acad. Sci. USA.

[21] Stuart T., Butler A., Hoffman P., Hafemeister C., Papalexi E., Mauck W.M., Hao Y., Stoeckius M., Smibert P., Satija R. (2019). Comprehensive integration of single-cell data. Cell.

[22] Ramilowski J.A., Goldberg T., Harshbarger J., Kloppmann E., Lizio M., Satagopam V.P., Itoh M., Kawaji H., Carninci P., Rost B. (2015). A draft network of ligand-receptor-mediated multicellular signalling in human. Nat. Commun..

[23] Krzywinski M., Schein J., Birol I., Connors J., Gascoyne R., Horsman D., Jones S.J., Marra M.A. (2009). Circos: An information aesthetic for comparative genomics. Genome Res..

[24] Korotkevich G., Sukhov V., Budin N., Shpak B., Artyomov M.N., Sergushichev A. (2021). Fast gene set enrichment analysis. bioRxiv.

[25] Wu T., Hu E., Xu S., Chen M., Guo P., Dai Z., Feng T., Zhou L., Tang W., Zhan L. (2021). Clusterprofiler 4.0: A universal enrichment tool for interpreting omics data. Innovation.

[26] Jassal B., Matthews L., Viteri G., Gong C., Lorente P., Fabregat A., Sidiropoulos K., Cook J., Gillespie M., Haw R. (2020). The reactome pathway knowledgebase. Nucleic Acids Res..

[27] Ernst J., Bar-Joseph Z. (2006). Stem: A tool for the analysis of short time series gene expression data. BMC Bioinform..

[28] Shin D.W., Kim J. (2020). The american joint committee on cancer 8th edition staging system for the pancreatic ductal adenocarcinoma: Is it better than the 7th edition?. Hepatobiliary Surg. Nutr..

[29] Louault K., Li R.R., DeClerck Y.A. (2020). Cancer-associated fibroblasts: Understanding their heterogeneity. Cancers.

[30] Werner S., Lutzkendorf J., Muller T., Muller L.P., Posern G. (2019). Mrtf-a controls myofibroblastic differentiation of human multipotent stromal cells and their tumour-supporting function in xenograft models. Sci. Rep..

[31] Labernadie A., Kato T., Brugues A., Serra-Picamal X., Derzsi S., Arwert E., Weston A., Gonzalez-Tarrago V., Elosegui-Artola A., Albertazzi L. (2017). A mechanically active heterotypic e-cadherin/n-cadherin adhesion enables fibroblasts to drive cancer cell invasion. Nat. Cell Biol..

[32] Pakshir P., Alizadehgiashi M., Wong B., Coelho N.M., Chen X., Gong Z., Shenoy V.B., McCulloch C.A., Hinz B. (2019). Dynamic fibroblast contractions attract remote macrophages in fibrillar collagen matrix. Nat. Commun..

[33] Davidson J., Shen Z., Gong X., Pollack J.R. (2018). Swi/snf aberrations sensitize pancreatic cancer cells to DNA crosslinking agents. Oncotarget.

[34] Liu T., Zhou L., Yang K., Iwasawa K., Kadekaro A.L., Takebe T., Andl T., Zhang Y. (2019). The beta-catenin/yap signaling axis is a key regulator of melanoma-associated fibroblasts. Signal Transduct. Target Ther..

[35] Garcia P.E., Scales M.K., Allen B.L., Pasca di Magliano M. (2020). Pancreatic fibroblast heterogeneity: From development to cancer. Cells.

[36] Zhang H., Kong Q., Wang J., Jiang Y., Hua H. (2020). Complex roles of camp-pka-creb signaling in cancer. Exp. Hematol. Oncol..

[37] Li G., Jiang Q., Xu K. (2019). Creb family: A significant role in liver fibrosis. Biochimie.

[38] Leung C.S., Yeung T.L., Yip K.P., Pradeep S., Balasubramanian L., Liu J., Wong K.K., Mangala L.S., Armaiz-Pena G.N., Lopez-Berestein G. (2014). Calcium-dependent fak/creb/tnnc1 signalling mediates the effect of stromal mfap5 on ovarian cancer metastatic potential. Nat. Commun..

[39] Jang I., Beningo K.A. (2019). Integrins, cafs and mechanical forces in the progression of cancer. Cancers.

[40] Ding J., Liu Y., Lai Y. (2020). Identifying mmp14 and col12a1 as a potential combination of prognostic biomarkers in pancreatic ductal adenocarcinoma using integrated bioinformatics analysis. PeerJ.

[41] Weniger M., Honselmann K.C., Liss A.S. (2018). The extracellular matrix and pancreatic cancer: A complex relationship. Cancers.

[42] Liu-Chittenden Y., Jain M., Gaskins K., Wang S., Merino M.J., Kotian S., Kumar Gara S., Davis S., Zhang L., Kebebew E. (2017). Rarres2 functions as a tumor suppressor by promoting beta-catenin phosphorylation/degradation and inhibiting p38 phosphorylation in adrenocortical carcinoma. Oncogene.

[43] Shin W.J., Zabel B.A., Pachynski R.K. (2018). Mechanisms and functions of chemerin in cancer: Potential roles in therapeutic intervention. Front. Immunol..

[44] Ruiz-Villalba A., Romero J.P., Hernandez S.C., Vilas-Zornoza A., Fortelny N., Castro-Labrador L., San Martin-Uriz P., Lorenzo-Vivas E., Garcia-Olloqui P., Palacio M. (2020). Single-cell rna sequencing analysis reveals a crucial role for cthrc1 (collagen triple helix repeat containing 1) cardiac fibroblasts after myocardial infarction. Circulation.

[45] Azuma T., Witke W., Stossel T.P., Hartwig J.H., Kwiatkowski D.J. (1998). Gelsolin is a downstream effector of rac for fibroblast motility. EMBO J..

[46] Elyada E., Bolisetty M., Laise P., Flynn W.F., Courtois E.T., Burkhart R.A., Teinor J.A., Belleau P., Biffi G., Lucito M.S. (2019). Cross-species single-cell analysis of pancreatic ductal adenocarcinoma reveals antigen-presenting cancer-associated fibroblasts. Cancer Discov..

[47] Crowe L.A.N., McLean M., Kitson S.M., Melchor E.G., Patommel K., Cao H.M., Reilly J.H., Leach W.J., Rooney B.P., Spencer S.J. (2019). S100a8 & s100a9: Alarmin mediated inflammation in tendinopathy. Sci. Rep..

[48] Wang S., Song R., Wang Z., Jing Z., Wang S., Ma J. (2018). S100a8/a9 in inflammation. Front. Immunol..

[49] Wang Y., Jing Y., Ding L., Zhang X., Song Y., Chen S., Zhao X., Huang X., Pu Y., Wang Z. (2019). Epiregulin reprograms cancer-associated fibroblasts and facilitates oral squamous cell carcinoma invasion via jak2-stat3 pathway. J. Exp. Clin. Cancer Res..

[50] Palmieri C., Roberts-Clark D., Assadi-Sabet A., Coope R.C., O’Hare M., Sunters A., Hanby A., Slade M.J., Gomm J.J., Lam E.W. (2003). Fibroblast growth factor 7, secreted by breast fibroblasts, is an interleukin-1beta-induced paracrine growth factor for human breast cells. J. Endocrinol..

[51] Hirakawa T., Yashiro M., Doi Y., Kinoshita H., Morisaki T., Fukuoka T., Hasegawa T., Kimura K., Amano R., Hirakawa K. (2016). Pancreatic fibroblasts stimulate the motility of pancreatic cancer cells through igf1/igf1r signaling under hypoxia. PLoS ONE.

[52] Tao J., Yang G., Zhou W., Qiu J., Chen G., Luo W., Zhao F., You L., Zheng L., Zhang T. (2021). Targeting hypoxic tumor microenvironment in pancreatic cancer. J. Hematol. Oncol..

[53] Li Y., Yu S., Li L., Chen J., Quan M., Li Q., Gao Y. (2020). Klf4-mediated upregulation of cd9 and cd81 suppresses hepatocellular carcinoma development via jnk signaling. Cell Death Dis..

[54] Koninger J., Giese N.A., di Mola F.F., Berberat P., Giese T., Esposito I., Bachem M.G., Buchler M.W., Friess H. (2004). Overexpressed decorin in pancreatic cancer: Potential tumor growth inhibition and attenuation of chemotherapeutic action. Clin. Cancer Res..

[55] Gold D.V., Stein R., Burton J., Goldenberg D.M. (2010). Enhanced expression of cd74 in gastrointestinal cancers and benign tissues. Int. J. Clin. Exp. Pathol..

[56] Zhang J.F., Hua R., Liu D.J., Liu W., Huo Y.M., Sun Y.W. (2014). Effect of cd74 on the prognosis of patients with resectable pancreatic cancer. Hepatobiliary Pancreat. Dis. Int..

[57] DiGiacomo V., Meruelo D. (2016). Looking into laminin receptor: Critical discussion regarding the non-integrin 37/67-kda laminin receptor/rpsa protein. Biol. Rev..

[58] Digiacomo V., Gando I.A., Venticinque L., Hurtado A., Meruelo D. (2015). The transition of the 37-kda laminin receptor (rpsa) to higher molecular weight species: Sumoylation or artifact?. Cell Mol. Biol. Lett..

[59] Novin A., Suhail Y., Ajeti V., Goyal R., Wali K., Seck A., Jackson A., Kshitiz (2021). Diversity in cancer invasion phenotypes indicates specific stroma regulated programs. Hum. Cell.

[60] Griss J., Viteri G., Sidiropoulos K., Nguyen V., Fabregat A., Hermjakob H. (2020). ReactomeGSA—Efficient multi-omics comparative pathway analysis. Mol. Cell. Proteom..

[61] Ganini C., Amelio I., Bertolo R., Bove P., Buonomo O.C., Candi E., Cipriani C., Di Daniele N., Juhl H., Mauriello A. (2021). Global mapping of cancers: The cancer genome atlas and beyond. Mol. Oncol..

[62] Karpinsky-Semper D., Volmar C.H., Brothers S.P., Slepak V.Z. (2014). Differential effects of the gbeta5-rgs7 complex on muscarinic m3 receptor-induced Ca^2+^ influx and release. Mol. Pharmacol..

[63] Ding T., Zhou Y., Long R., Chen C., Zhao J., Cui P., Guo M., Liang G., Xu L. (2019). Dusp8 phosphatase: Structure, functions, expression regulation and the role in human diseases. Cell Biosci..

[64] Gao Y., Wang J., Fan G. (2012). Nprl2 is an independent prognostic factor of osteosarcoma. Cancer Biomark..

[65] Peng H.Y., Yu Q.F., Shen W., Guo C.M., Li Z., Zhou X.Y., Zhou N.J., Min W.P., Gao D. (2016). Knockdown of elmo3 suppresses growth, invasion and metastasis of colorectal cancer. Int. J. Mol. Sci..

[66] Jin G., Hong W., Guo Y., Bai Y., Chen B. (2020). Molecular mechanism of pancreatic stellate cells activation in chronic pancreatitis and pancreatic cancer. J. Cancer.

[67] Shimizu K. (2008). Pancreatic stellate cells: Molecular mechanism of pancreatic fibrosis. J. Gastroenterol. Hepatol..

[68] Martinez-Bosch N., Vinaixa J., Navarro P. (2018). Immune evasion in pancreatic cancer: From mechanisms to therapy. Cancers.

[69] Zeng S., Pottler M., Lan B., Grutzmann R., Pilarsky C., Yang H. (2019). Chemoresistance in pancreatic cancer. Int. J. Mol. Sci..

[70] Sarr A., Bre J., Um I.H., Chan T.H., Mullen P., Harrison D.J., Reynolds P.A. (2019). Genome-scale crispr/cas9 screen determines factors modulating sensitivity to protide nuc-1031. Sci. Rep..

[71] He P., Yang J.W., Yang V.W., Bialkowska A.B. (2018). Kruppel-like factor 5, increased in pancreatic ductal adenocarcinoma, promotes proliferation, acinar-to-ductal metaplasia, pancreatic intraepithelial neoplasia, and tumor growth in mice. Gastroenterology.

[72] Mori A., Moser C., Lang S.A., Hackl C., Gottfried E., Kreutz M., Schlitt H.J., Geissler E.K., Stoeltzing O. (2009). Up-regulation of kruppel-like factor 5 in pancreatic cancer is promoted by interleukin-1beta signaling and hypoxia-inducible factor-1alpha. Mol. Cancer Res..

[73] Kabacaoglu D., Ruess D.A., Ai J., Algul H. (2019). Nf-kappab/rel transcription factors in pancreatic cancer: Focusing on rela, c-rel, and relb. Cancers.

[74] Xiong J., Li Y., Tan X., Fu L. (2020). Small heat shock proteins in cancers: Functions and therapeutic potential for cancer therapy. Int. J. Mol. Sci..

[75] Uhlen M., Fagerberg L., Hallstrom B.M., Lindskog C., Oksvold P., Mardinoglu A., Sivertsson A., Kampf C., Sjostedt E., Asplund A. (2015). Proteomics. Tissue-based map of the human proteome. Science.

[76] Sun W., Hu C., Wang T., Wang J., Zhang J., Gao F., Ou Q., Tian H., Jin C., Xu J. (2021). Glia maturation factor beta as a novel biomarker and therapeutic target for hepatocellular carcinoma. Front. Oncol..

[77] Ling B., Watt K., Banerjee S., Newsted D., Truesdell P., Adams J., Sidhu S.S., Craig A.W.B. (2017). A novel immunotherapy targeting mmp-14 limits hypoxia, immune suppression and metastasis in triple-negative breast cancer models. Oncotarget.

[78] Claesson-Welsh L. (2020). How the matrix metalloproteinase mmp14 contributes to the progression of colorectal cancer. J. Clin. Investig..

[79] Mueller M.M., Fusenig N.E. (2004). Friends or foes—Bipolar effects of the tumour stroma in cancer. Nat. Rev. Cancer.

[80] Kwa M.Q., Herum K.M., Brakebusch C. (2019). Cancer-associated fibroblasts: How do they contribute to metastasis?. Clin. Exp. Metastasis.

[81] Sahai E., Astsaturov I., Cukierman E., DeNardo D.G., Egeblad M., Evans R.M., Fearon D., Greten F.R., Hingorani S.R., Hunter T. (2020). A framework for advancing our understanding of cancer-associated fibroblasts. Nat. Rev. Cancer.

[82] Okabe Y., Medzhitov R. (2016). Tissue biology perspective on macrophages. Nat. Immunol..

[83] Colegio O.R., Chu N.Q., Szabo A.L., Chu T., Rhebergen A.M., Jairam V., Cyrus N., Brokowski C.E., Eisenbarth S.C., Phillips G.M. (2014). Functional polarization of tumour-associated macrophages by tumour-derived lactic acid. Nature.

[84] Sanchez L.R., Borriello L., Entenberg D., Condeelis J.S., Oktay M.H., Karagiannis G.S. (2019). The emerging roles of macrophages in cancer metastasis and response to chemotherapy. J. Leukoc. Biol..

