# Supplementary material for: Metastatic Transition of Pancreatic Ductal Cell Adenocarcinoma Is Accompanied by the Emergence of Pro-Invasive Cancer-Associated Fibroblasts
Source: Cancers (Basel). 2022 Apr 28;14(9):2197. doi: 10.3390/cancers14092197 (PMC9104173; doi:10.3390/cancers14092197)
Supplement: Supplementary file 1 [file cancers-14-02197-s001.zip › cancers-1675343-supplementary.pdf]

Supplementary Materials: Metastatic Transition of Pancreatic Ductal Cell Adenocarcinoma is accompanied by Emergence of Pro-Invasive Cancer associated Fibroblasts

Shaofei Liu, Yasir Suhail, Ashkan Novin and Kshitiz

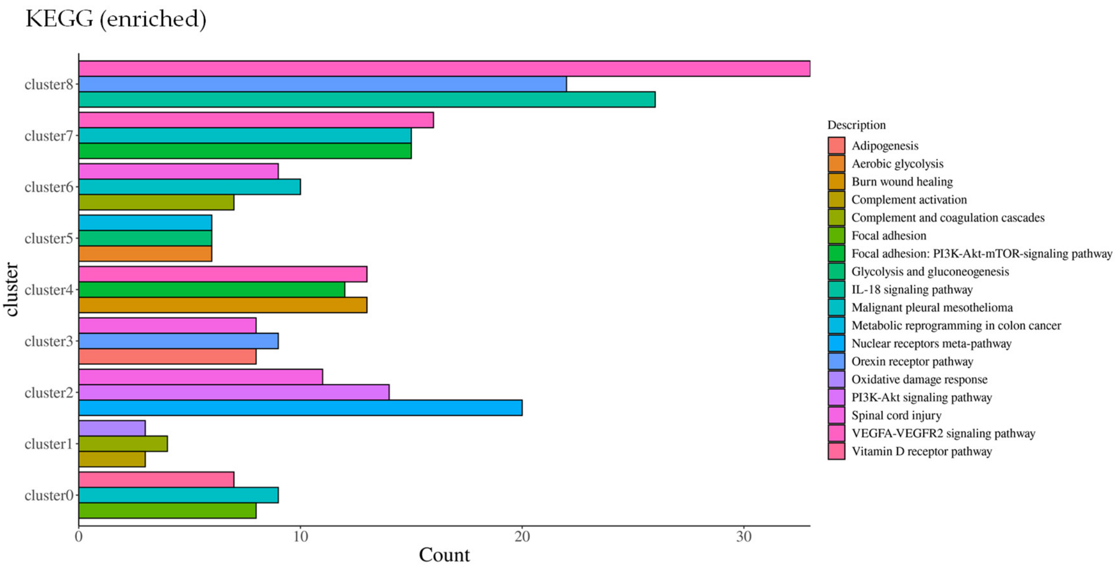

Figure S1. Kegg pathway activation in different fibroblast clusters identified in pooled PDAC data for biological processes; Each term is obtained by differential gene expression analysis (log2fc ≥ 0.5, *p*-value ≤ 0.05).

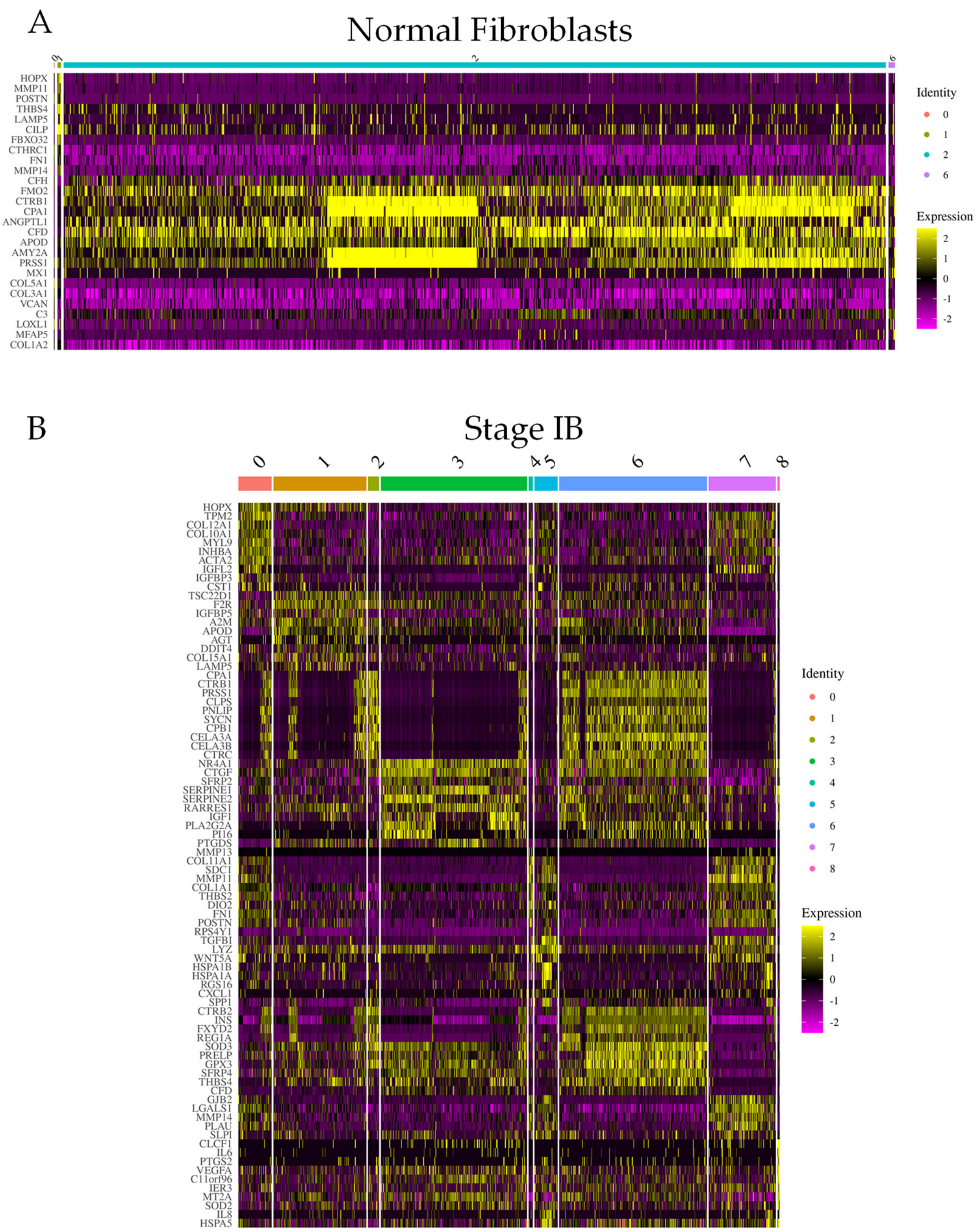

Figure S2. Heatmap showing top 10 genes as markers for fibroblasts from normal pancreas (**A**), and fibroblasts in stage IB (**B**), compared to all other fibroblasts; Each fibroblast cluster in this stage described in Figure 1A is shown in a different section, color coded (top).

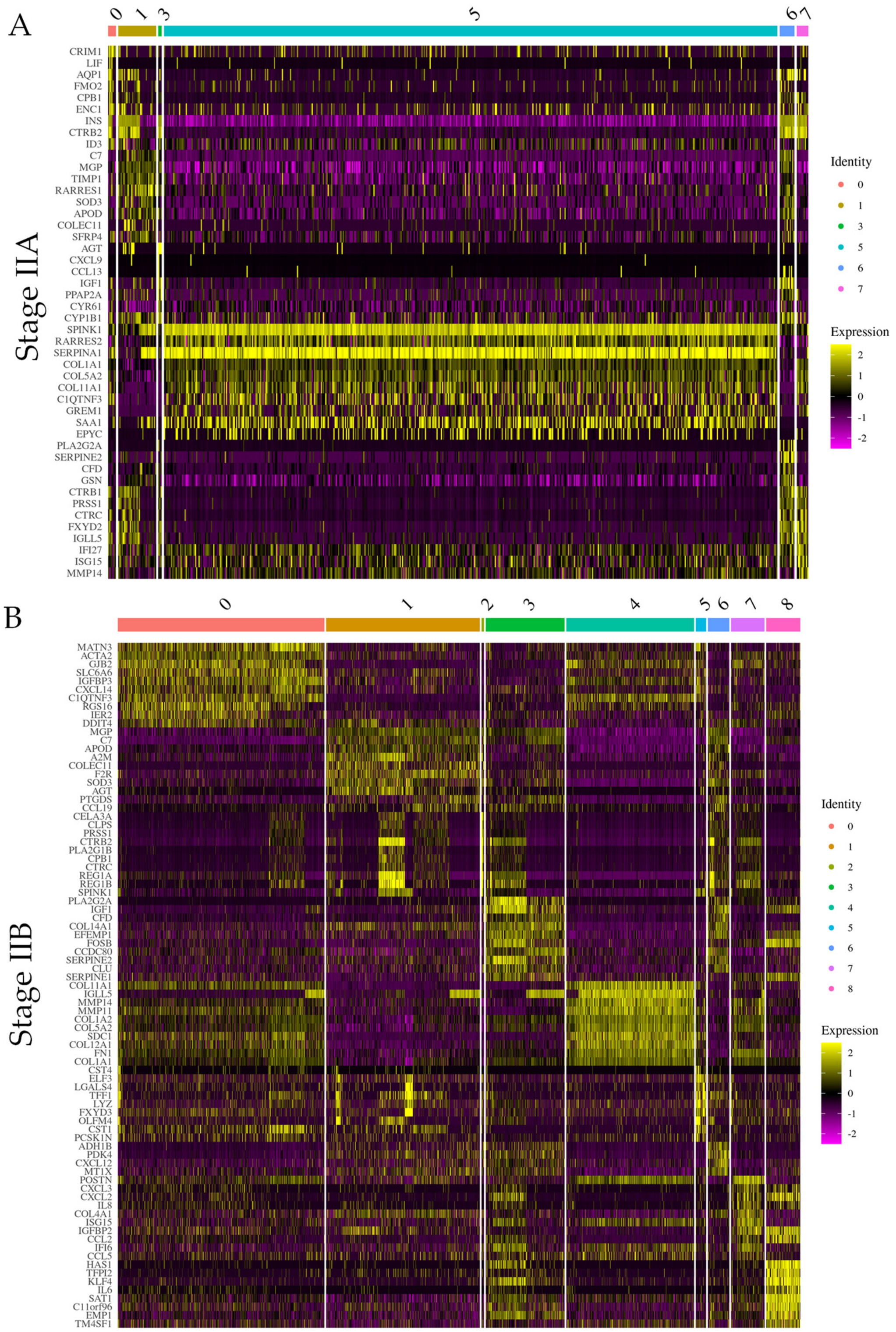

Figure S3. Heatmap showing top 10 genes as markers for fibroblasts from stage IIA (**A**), and fibroblasts in stage IIB (**B**), compared to all other fibroblasts; Each fibroblast cluster in this stage described in Figure 1A is shown in a different section, color coded (top).

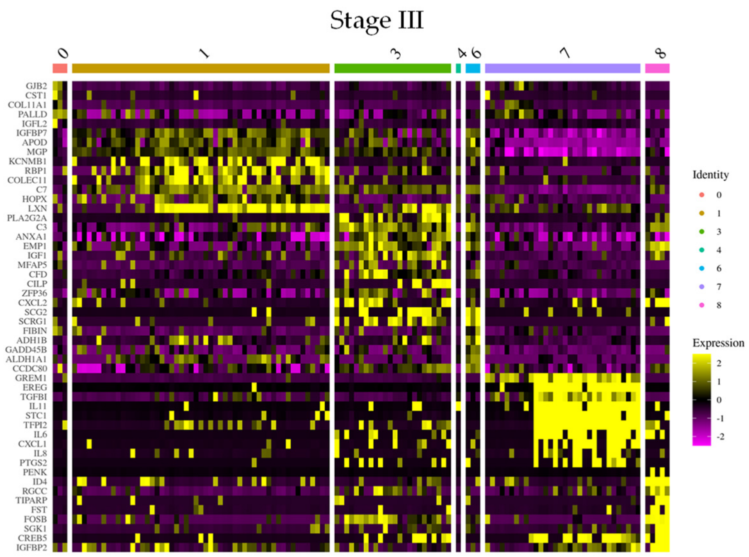

Figure S4. Heatmap showing top 10 genes as markers for fibroblasts from fibroblasts in stage III, compared to all other fibroblasts; Each fibroblast cluster in this stage described in Figure 1A is shown in a different section, color coded (top).

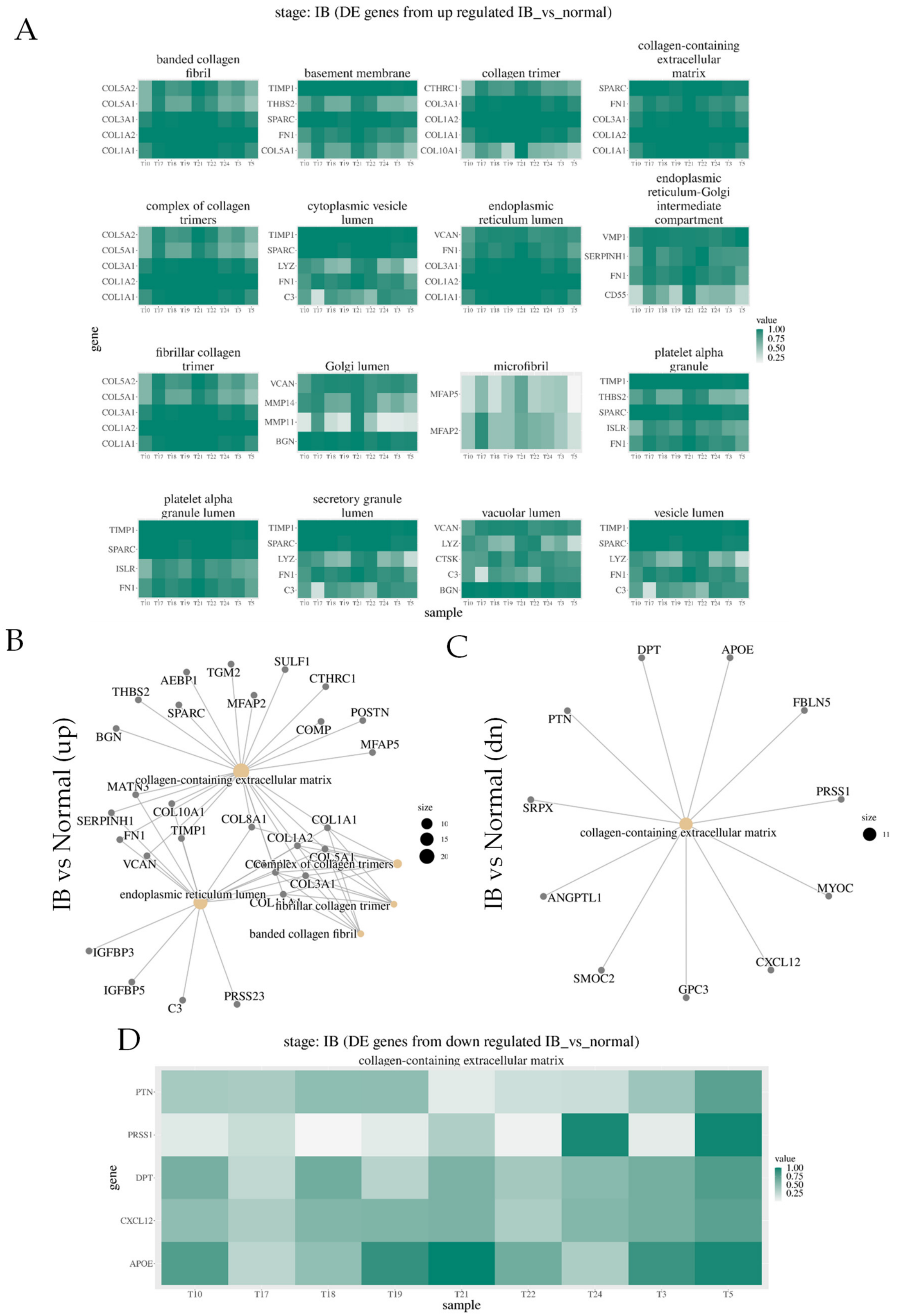

Figure S5. Sample-wise gene expression in top activated gene ontology in stage IB vs normal fibroblasts. (**A**) Top 5 genes in GOs activated in stage IB vs normal (see Figure 2A), with chosen gene-sets and their genes shown as network (**B**). (**C**–**D**) Top 5 genes in GOs inactivated in stage IB vs normal (see Figure 2A), with chosen gene-sets and their genes shown as network (**D**). The top 5 selected genes are based on log2(fold change) for both positive, and negative gene-set activation.

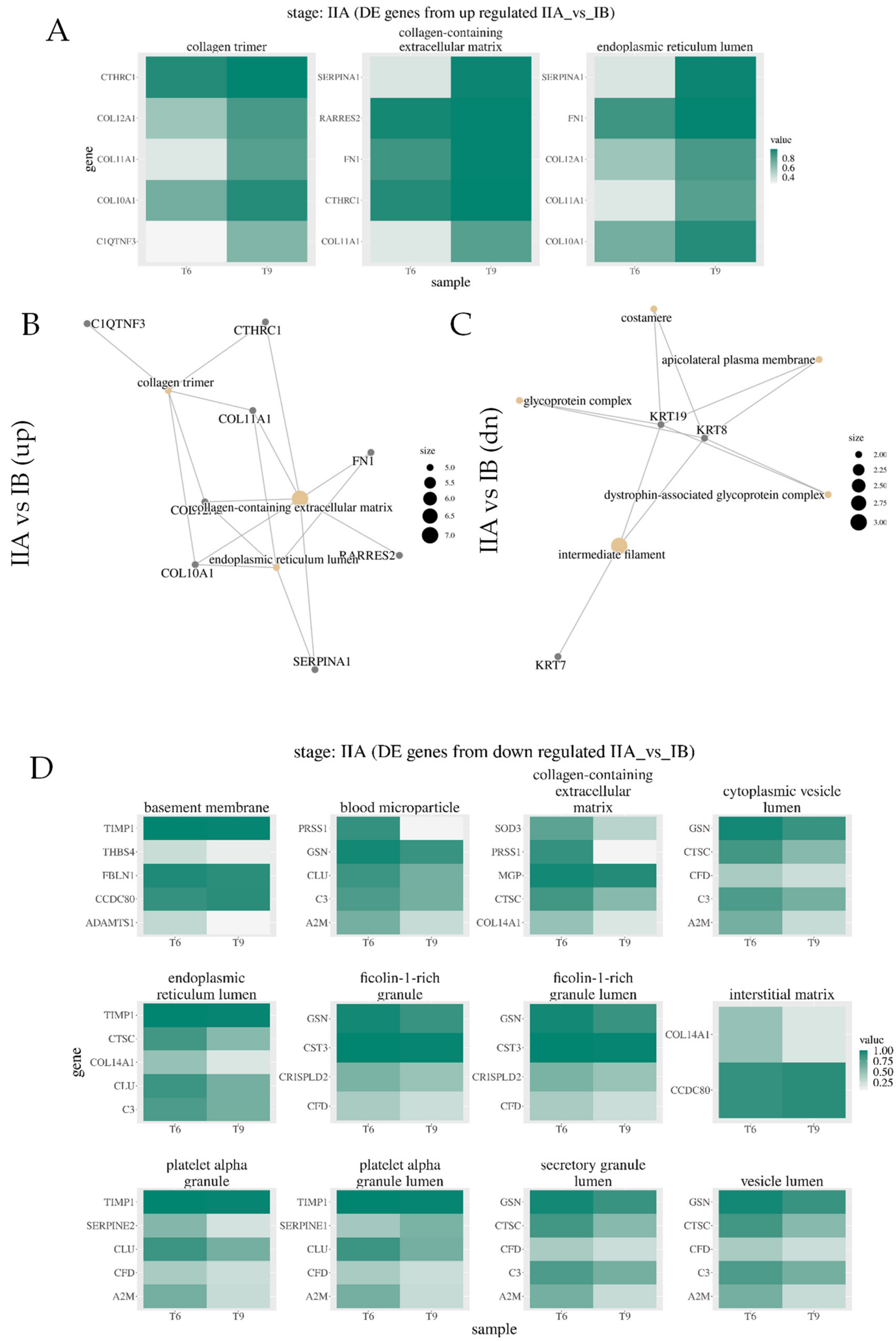

Figure S6. Sample-wise gene expression in top activated gene ontology in fibroblasts in stage IIA vs IB. (**A**) Top 5 genes in GOs activated in stage IIA vs IB (see Figure 2C), with chosen gene-sets and their genes shown as network (**B**). (**C**) Top 5 genes in GOs inactivated in stage IIA vs IB (see Figure 2C), with chosen gene-sets and their genes shown as network (D). The top 5 selected genes are based on log2(fold change) for both positive, and negative gene-set activation.

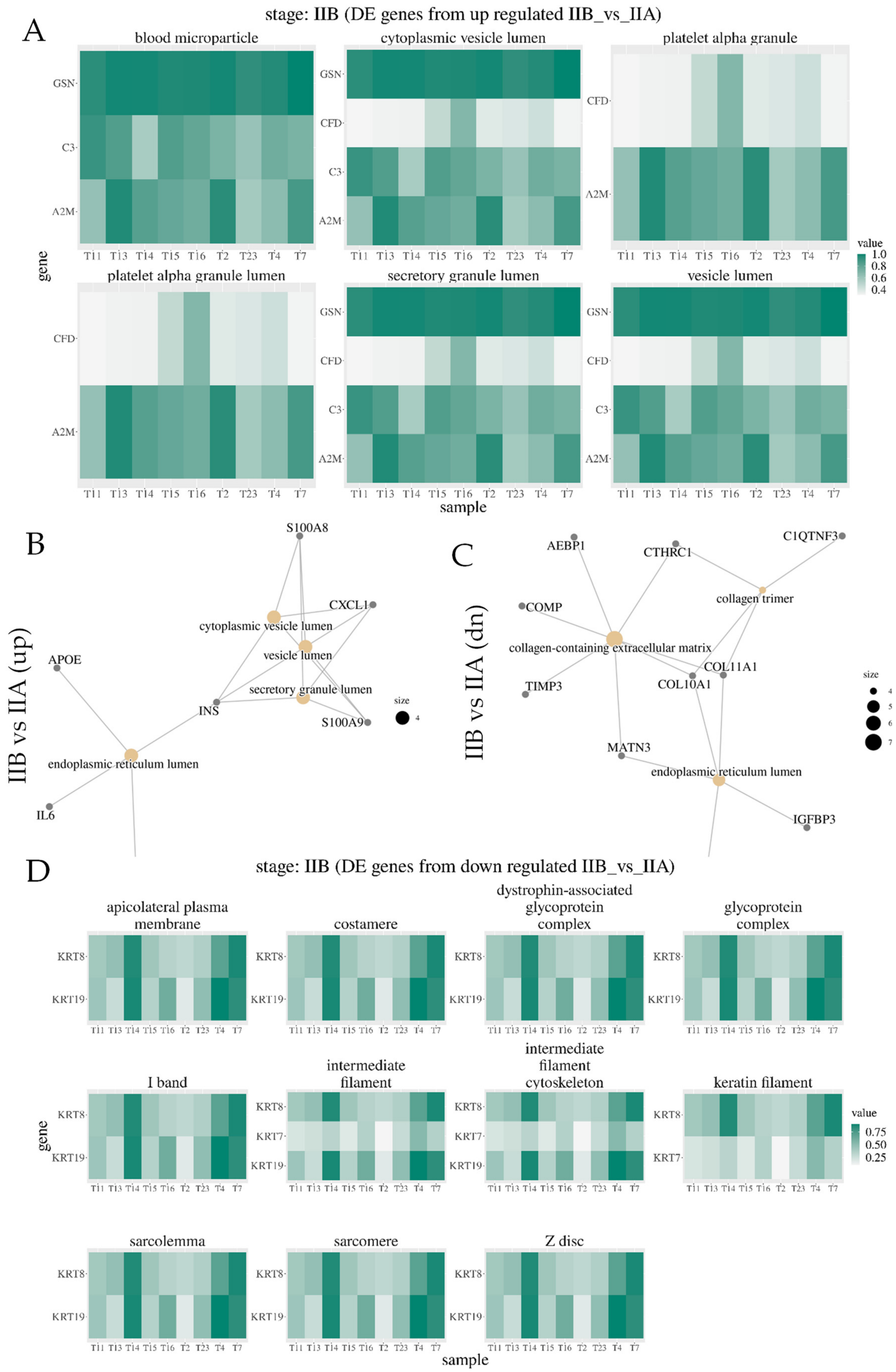

Figure S7. Sample-wise gene expression in top activated gene ontology in fibroblasts in stage IIB vs IIA. (**A**) Top 5 genes in GOs activated in stage IIB vs IIA (see Figure 2E), with chosen gene-sets and

their genes shown as network (**B**). (**C**) Top 5 genes in GOs inactivated in stage IIB vs IIA (see Figure 2G), with chosen gene-sets and their genes shown as network (**D**).

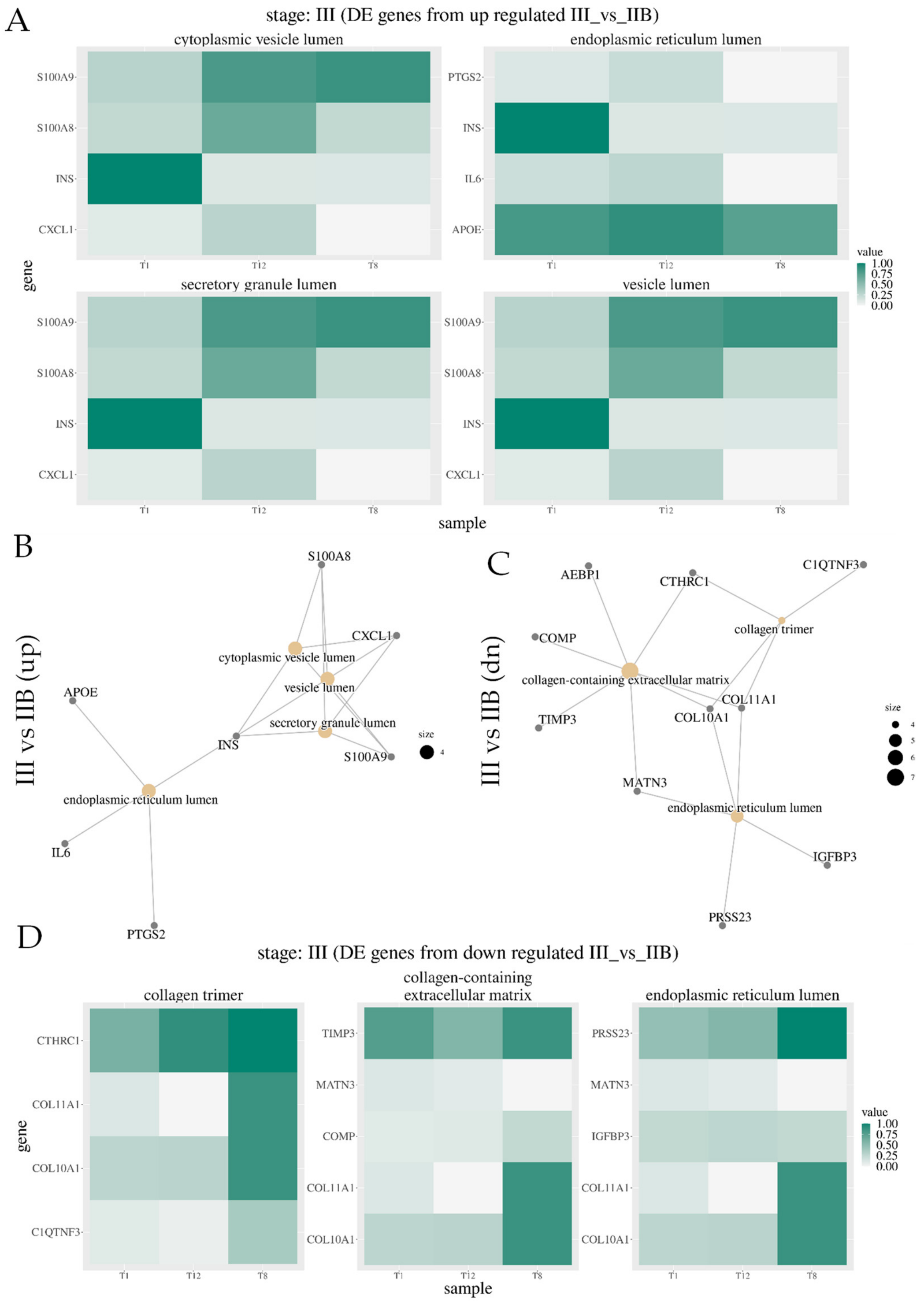

Figure S8. Sample-wise gene expression in top activated gene ontology in fibroblasts in stage III vs IIB. (**A**) Top 5 genes in GOs activated in stage III vs IIB (see Figure 2I), with chosen gene-sets and their genes shown as network (**B**). (**C**) Top 5 genes in GOs inactivated in stage III vs IIB (see Figure 2I), with chosen gene-sets and their genes shown as network (**D**).

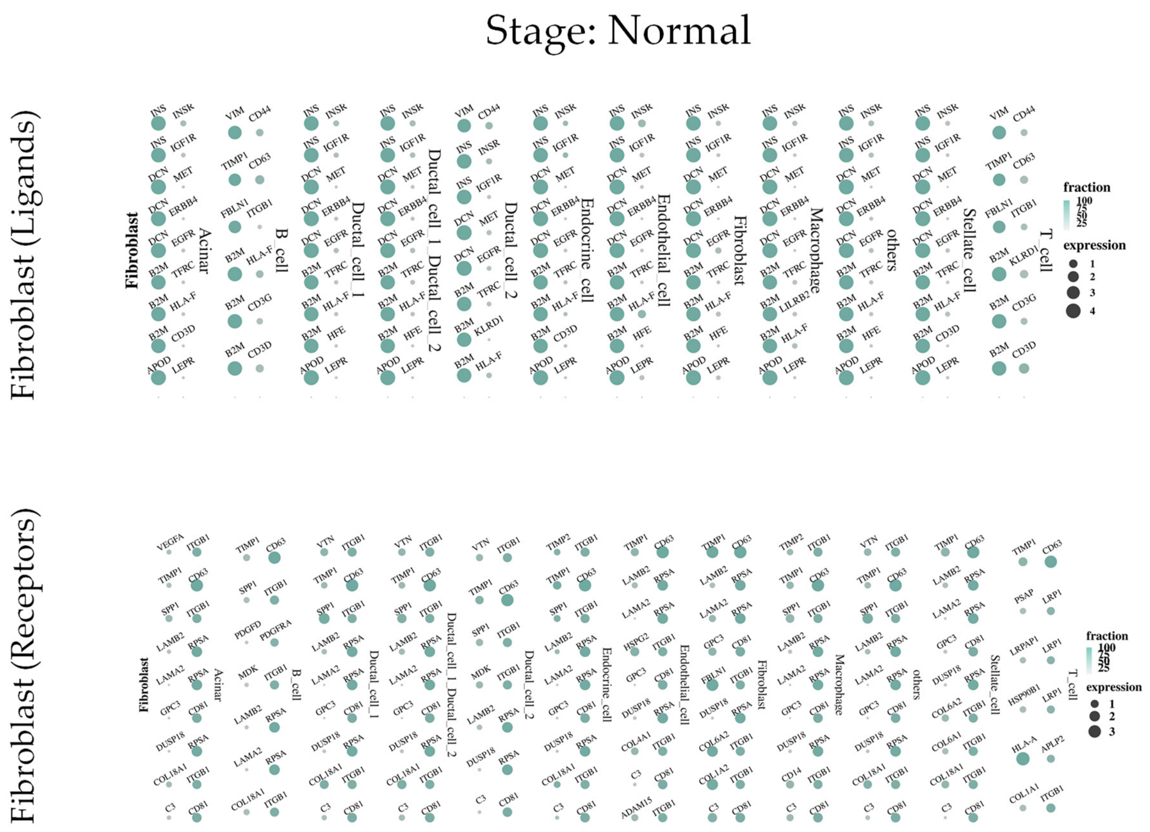

Figure S9. Ligand-receptor interaction between fibroblasts and other cell types in normal pancreas. Bubble plots showing genes encoding ligands, and receptors in fibroblasts from normal pancreas and their putative receptors, and ligands expression respectively in other cell types (see Figure 3A–B).

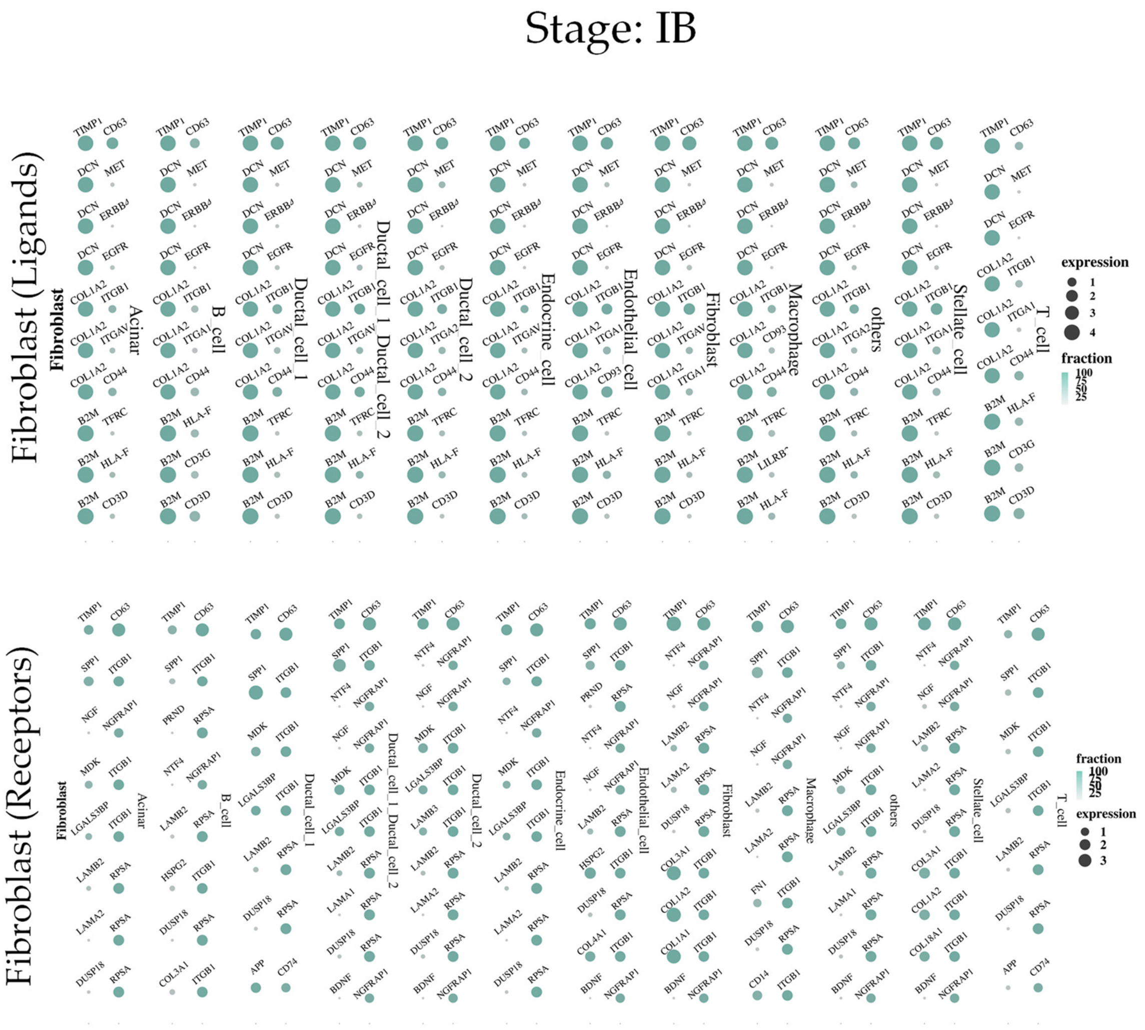

Figure S10. Ligand-receptor interaction between fibroblasts and other cell types in fibroblasts from stage IB. Bubble plots showing genes encoding ligands, and receptors in fibroblasts from stage IB and their putative receptor and ligand expression respectively in other cell types (see Figure 3C–D).

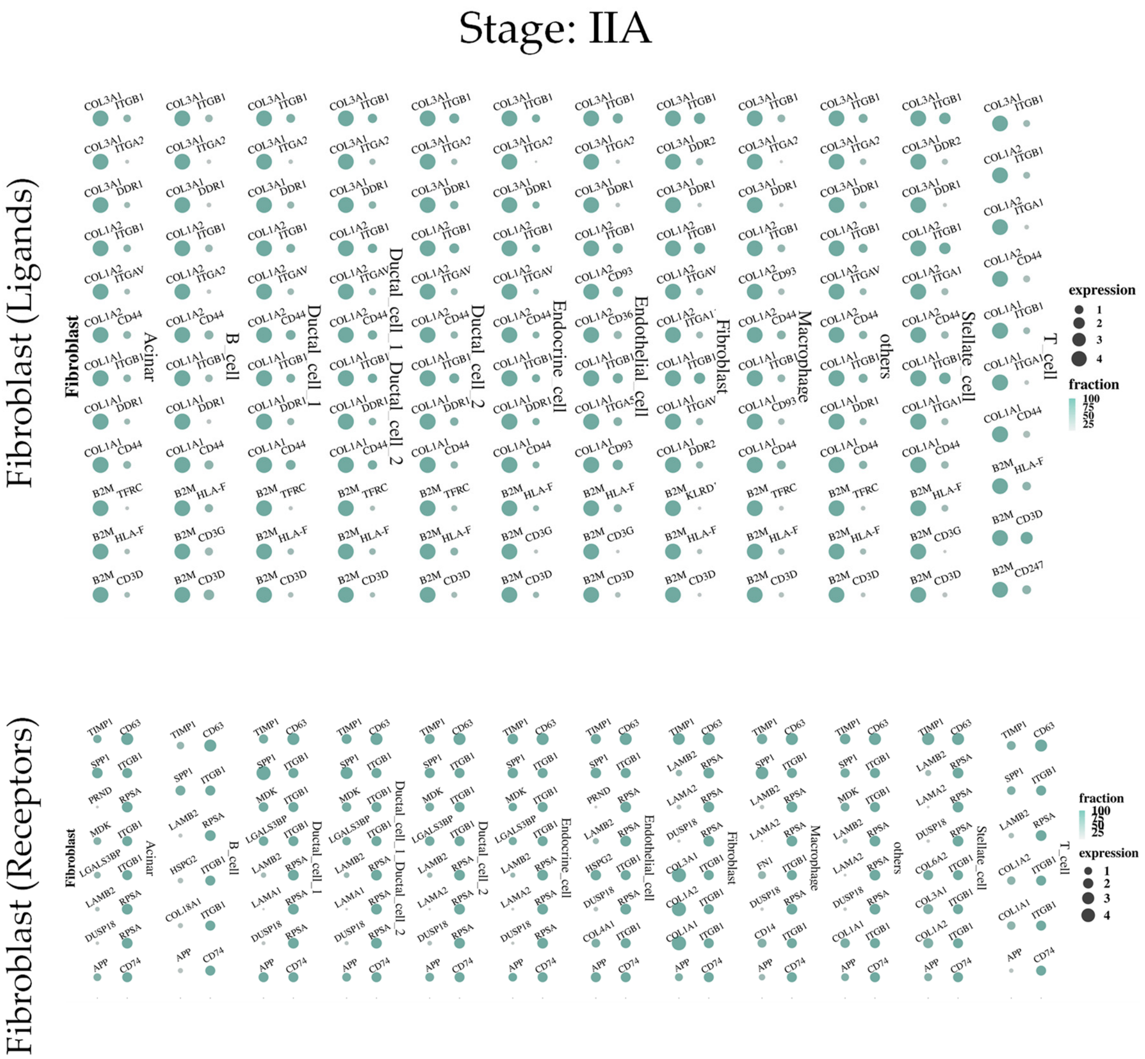

Figure S11. Ligand-receptor interaction between fibroblasts and other cell types in fibroblasts from stage IIA. Bubble plots showing genes encoding ligands, and receptors in fibroblasts from stage IIA and their putative receptor and ligand expression respectively in other cell types (see Figure 3E–F).

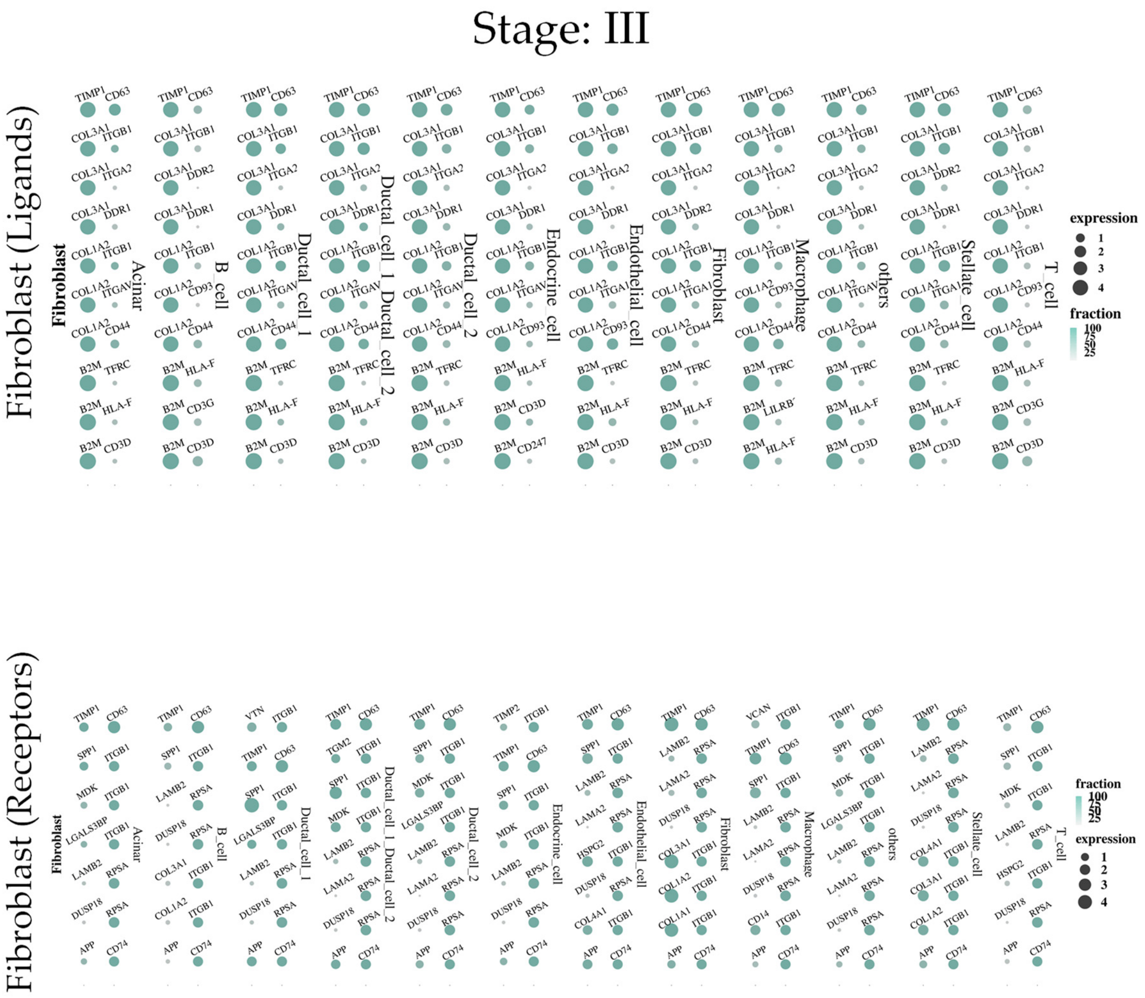

Figure S12. Ligand-receptor interaction between fibroblasts and other cell types in fibroblasts from stage IIB. Bubble plots showing genes encoding ligands, and receptors in fibroblasts from stage IIB and their putative receptor and ligand expression respectively in other cell types (see Figure 3G–H).

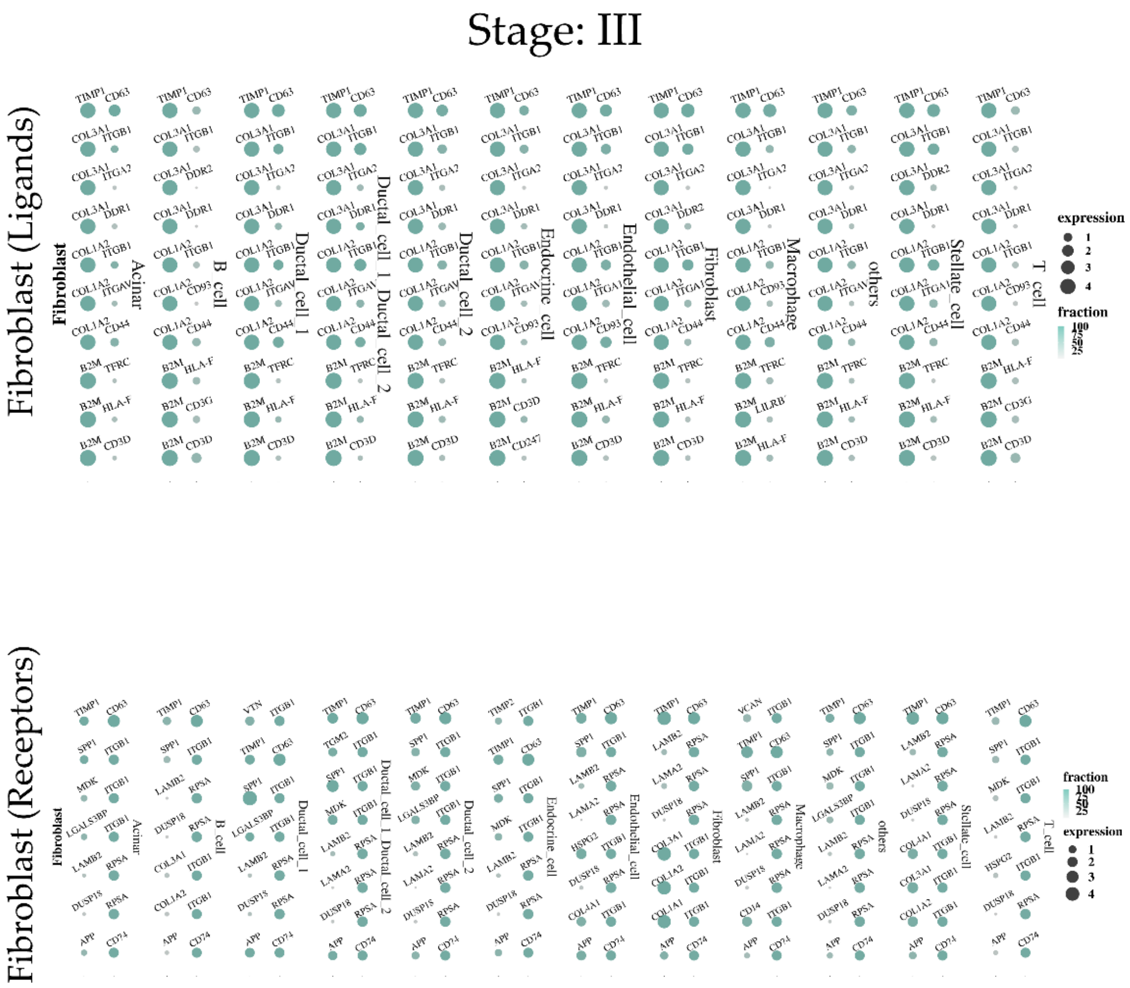

Figure S13. Ligand-receptor interaction between fibroblasts and other cell types in fibroblasts from stage III. Bubble plots showing genes encoding ligands, and receptors in fibroblasts from stage III and their putative receptor and ligand expression respectively in other cell types (see Figure 3I–J).

Leading Edge Genes: N1 vs N0 stages and ELIdn enrichment					
GPANK1	RGS7	ARL3	IRF2BP1	PCYOX1L	GALT
MSTO1	DUSP8	RNASEH2C	LRRC73	WDR83	ALDH5A1
SLC25A1	NRPL2	ELMO3	MDH1B	FAM98B	RGS9
NTPCR	ANKRD16	TIMM22	CPT2	COX10	MANSC1
CFAP46	B4GALT6	DHDH	CFAP53	MFSD2A	OARD1
FITM2	PPM1E	MCAT	UCHL1	PPP6C	HR
CCNB3	HDDC2	WDR17	IFT22	GMDS	CDK20
DGCR8	PUS3	GFRA3	SLC18A2	TMEM232	PLEKHD1
FAM210B	OXA1L	MMUT	NANS	PSMG3	SYT9
GNPDA2	TIMP1	SMOC2	TRPM3	HDDC3	SETD6
MDFI	GALNT16	MLLT11	GDNF	FAM8A1	ALAS1

Figure S14. Leading edge genes in GSEA analysis showing enrichment of ELIdn genes among differentially expressed gene-sets in TCGA PDAC patients with ($N = 1$ in TNM score), or without ($N = 0$ in TNM score) lymph node metastasis.

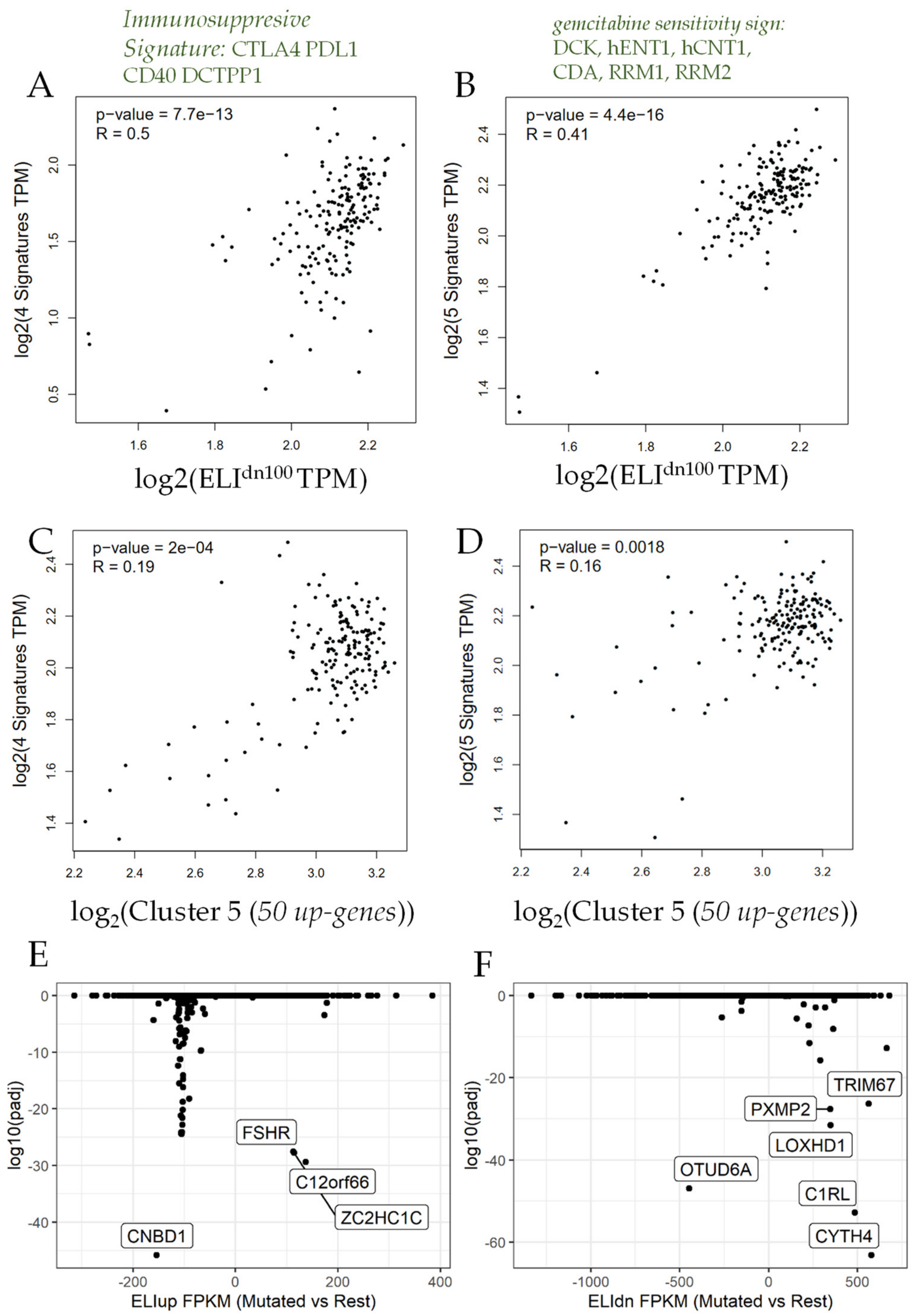

Figure S15. Gepia based gene signature correlation analysis for top 50 ELIdn genes for (**A**) key immunosuppressive genes, and (**B**) drug sensitivity genes for gemcitabine. Gepia2 based gene signature correlation analysis for top 50 up-regulated genes in cluster 5 (representing most fibroblasts in in stage IIA) for (**C**) key immunosuppressive genes, and (**D**) drug sensitivity genes for gemcitabine. R is calculated using Pearson's Correlation Analysis, *p*-value shown in the panels. (**E–F**) Correlation of tumor mutations to the gene expression of ELI^{up} (**E**), and ELI^{dn} (**F**) genes in TCGA pancreatic tumor samples; selected genes with mutations annotated.

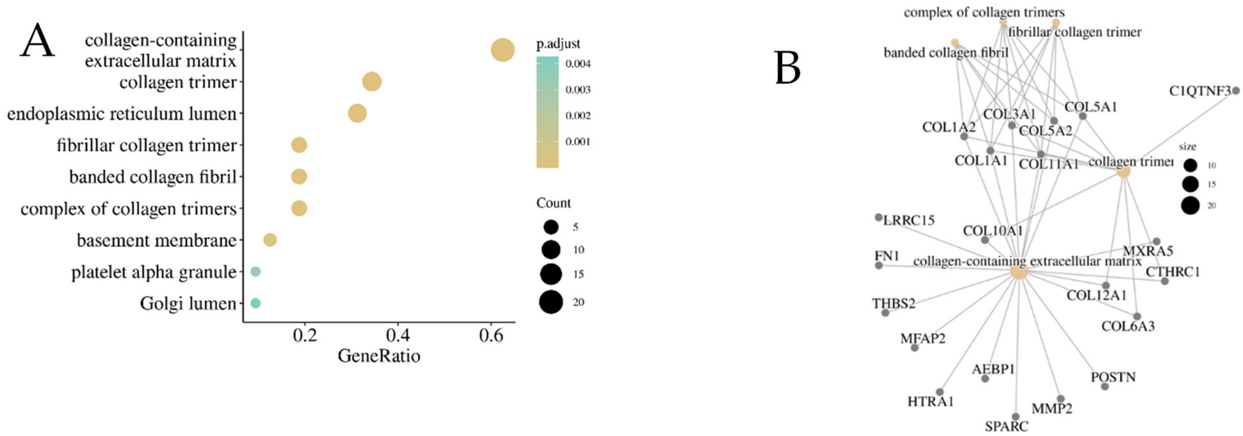

Figure S16. Gene ontologies activated in PDAC fibroblast subclass cluster 4. (**A**) Activated GOs in cluster 4 fibroblasts identified in Figure 1A compared to all other fibroblasts, with genes-GO network (log2(fc) ≥ 1, *p*-value ≤ 0.05) shown in (**B**). .
